# Analysis of Evolutionary Conservation, Expression Level, and Genetic Association at a Genome-wide Scale Reveals Heterogeneity Across Polygenic Phenotypes

**DOI:** 10.1093/molbev/msae115

**Published:** 2024-06-12

**Authors:** Ann-Sophie Giel, Jessica Bigge, Johannes Schumacher, Carlo Maj, Pouria Dasmeh

**Affiliations:** Centre for Human Genetics, Marburg University, Marburg, Germany; Centre for Human Genetics, Marburg University, Marburg, Germany; Centre for Human Genetics, Marburg University, Marburg, Germany; Centre for Human Genetics, Marburg University, Marburg, Germany; Centre for Human Genetics, Marburg University, Marburg, Germany; Department of Chemistry and Chemical Biology, Harvard University, Cambridge, MA, USA; Institute for Evolutionary Biology and Environmental Studies, University of Zurich, Zurich, Switzerland

**Keywords:** complex traits, GWAS, evolution, schizophrenia, coronary artery disease

## Abstract

Understanding the expression level and evolutionary rate of associated genes with human polygenic diseases provides crucial insights into their disease-contributing roles. In this work, we leveraged genome-wide association studies (GWASs) to investigate the relationship between the genetic association and both the evolutionary rate (d*N*/d*S*) and expression level of human genes associated with the two polygenic diseases of schizophrenia and coronary artery disease. Our findings highlight a distinct variation in these relationships between the two diseases. Genes associated with both diseases exhibit a significantly greater variance in evolutionary rate compared to those implicated in monogenic diseases. Expanding our analyses to 4,756 complex traits in the GWAS atlas database, we unraveled distinct trait categories with a unique interplay among the evolutionary rate, expression level, and genetic association of human genes. In most polygenic traits, highly expressed genes were more associated with the polygenic phenotypes compared to lowly expressed genes. About 69% of polygenic traits displayed a negative correlation between genetic association and evolutionary rate, while approximately 30% of these traits showed a positive correlation between genetic association and evolutionary rate. Our results demonstrate the presence of a spectrum among complex traits, shaped by natural selection. Notably, at opposite ends of this spectrum, we find metabolic traits being more likely influenced by purifying selection, and immunological traits that are more likely shaped by positive selection. We further established the polygenic evolution portal (evopolygen.de) as a resource for investigating relationships and generating hypotheses in the field of human polygenic trait evolution.

## Introduction

Investigating the evolutionary rate of human disease genes is crucial for understanding the genetic basis of diseases and their evolution over time (Cai et al. 2009; Podder and Ghosh 2010; Chakraborty et al. 2016; Benton et al. 2021). This approach can provide insights into the fundamental questions in evolutionary medicine, such as the evolutionary mechanisms and biological properties of genes and gene regulatory networks that contribute to the persistence of disease variants within human populations (Priedigkeit et al. 2015; Di et al. 2021). This is particularly important for polygenic and complex diseases (Spataro et al. 2017), which result from the contribution of multiple genes. The genetic basis of such diseases is more complex than monogenic diseases (Frazer et al. 2009; Albert and Kruglyak 2015), and we often lack a systematic understanding of the evolutionary rate of the genes associated with such diseases.

There have been conflicting findings from earlier studies on the evolutionary rate of genes implicated in human diseases. Some studies have suggested that human disease genes are more evolutionarily conserved than other genes, indicating that mutations in disease-causing genes are more likely to be deleterious compared to nondisease genes (López-Bigas and Ouzounis 2004). Other works have shown that human disease genes have a higher rate of evolution, quantified by *dN*/*d*S ratio (i.e. the ratio of nonsynonymous substitution rate to synonymous substitution rate), compared to nondisease genes (Smith and Eyre-Walker 2003). This observation might indicate that human disease genes are subject to a weaker purifying selection, particularly genes that are associated with complex and polygenic diseases, compared to nondisease genes (Cai et al. 2009). One potential explanation for these conflicting findings is that the association between susceptibility genes and evolutionary rate can be trait-specific and not generalizable, as different traits may have experienced different selection pressures (Rosenberg et al. 2019; Song et al. 2021). For example, neurodevelopmental disorders may be under a strong evolutionary selection as they are associated with reduced fecundity (Power et al. 2013), whereas traits that arise later in life, such as neurodegenerative diseases, may be more neutral with respect to evolutionary pressure (Fox 2018). Additionally, genetic factors such as pleiotropy (Watanabe et al. 2019a) may affect the comparison of the evolutionary rate of disease and nondisease genes, as genes associated with diseases may also affect nondisease traits and vice versa.

Here, we argue that constructing a single set of genes associated with complex diseases and comparing them with genes implicated in monogenic diseases does not consider the polygenic nature of many complex traits in human. Complex diseases are often caused by multiple genetic and environmental factors, and the contribution of each factor may vary among individuals and populations. It is then difficult to define a single set of genes associated with polygenic diseases that would be comprehensive and representative of all individuals with different diseases. Furthermore, it is impossible to discuss the evolutionary rate of genes without considering their expression level since the expression level is the foremost determinant of evolutionary rate; with highly expressed genes exhibiting a slower rate of evolution, compared to lowly expressed genes (Drummond and Wilke 2008; Dasmeh et al. 2017).

We propose an alternative approach to study the evolutionary rate and expression level of complex diseases in a trait-specific manner. Our approach leverages genome-wide association studies (GWASs) and the aggregated impact of genetic variations on individual genes, allowing us to compare the evolutionary rate and expression level of disease and nondisease genes. To accomplish this, we utilized MAGMA (de Leeuw et al. 2015), an approach that measures the association between a gene set and a trait using GWAS summary statistics. MAGMA quantifies this association using a *z-*score that compares the observed association of a set of genetic markers within a gene set to the expected association of the same number of markers randomly selected from across the genome. A positive *z-*score indicates enrichment of the gene set for the trait, while a negative *z-*score indicates depletion of the gene set for the trait.

By employing this trait-specific approach, we tackle three key questions. Firstly, we explore the relationship between evolutionary rate and the expression level of genes associated with polygenic diseases. We investigate these relationships in the two highly polygenic diseases, schizophrenia and coronary artery disease, with a low genetic correlation. We then extend our analyses to 4,756 complex traits within the GWAS atlas database (de Leeuw et al. 2015; Watanabe et al. 2019a). Secondly, we compare the evolutionary rate of highly associated genes to either schizophrenia or coronary artery disease with the genes implicated in monogenic diseases. Finally, we examine how the relationship between genetic association and expression level varies across different tissues. Our study reveals distinct categories of complex traits, with a unique interplay between genetic association, evolutionary rate, and gene expression.

## Results

### The Relationship Between Genetic Association, Expression Level, and Evolutionary Rate is Trait-specific

In our first analysis, we focused on finding the differences in the evolutionary rate and expression level of associated and nonassociated genes to schizophrenia and coronary artery disease. We selected these polygenic diseases specifically due to their low genetic correlation (*R*_g_ ∼0.03, calculated by LD score regression; see Methods). This low genetic correlation ensures that the correlation between genetic association and either evolutionary rate or expression level is minimally influenced by the same genes contributing to both diseases, reducing potential bias. We calculated the association of human genes to both polygenic diseases using MAGMA (see Methods; supplementary tables S1, Supplementary Material online) and selected 1,000 genes with the highest and the lowest association to these diseases for our comparisons. These were genes with the highest and lowest MAGMA *z-*scores for each disease (see Methods).

In our study, we employed the d*N*/d*S* metric as the evolutionary rate of human genes, focusing on the strength and mode of natural selection. In brief, d*N*/d*S* represents the ratio of the rate of nonsynonymous substitutions to that of synonymous substitutions. When the normalized rate of nonsynonymous substitutions (d*N*) surpasses that of synonymous substitutions (d*S*), it signifies that a protein is undergoing positive selection as nonsynonymous mutations are fixated with a higher rate. Conversely, when d*N*/d*S* < 1, it typically suggests that the protein is evolving under negative (purifying) selection, resulting in a higher evolutionary conservation. It is important to note that although d*N*/d*S* is commonly perceived as the evolutionary rate, a gene can evolve with a high d*N*/d*S* but with a low rate of nucleotide substitutions (Buschiazzo et al. 2012). This ratio thus primarily serves as a measure to assess the relative impact of natural selection on protein-coding sequences.

**Fig. 1.**
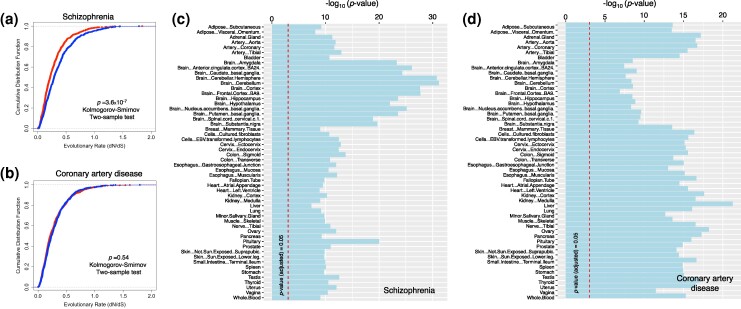
The evolutionary rate and expression level of highly and lowly associated genes with schizophrenia and coronary artery disease. a, b) The cumulative distribution functions (CDF) of the evolutionary rate (dN/dS) of 1000 genes with the highest association (in red), and 1000 genes with the lowest association (in blue) for schizophrenia (panel a), and coronary artery disease (panel b). The *P*-values in both panels are calculated from a two-sample Kolmogorov-Smirnov test. c, d) Tissue-specific average expression level (in units of transcripts per million mapped reads; number of RNA transcript copies per million mapped reads) of 1000 genes with the highest and lowest associations for schizophrenia (panel c), and coronary artery disease (panel d). The horizontal dashed lines in panels c and d correspond to the Bonferroni-corrected *P*-value, i.e. -log10(0.05/54) = 3.03.

The evolutionary rate of genes with the highest association was significantly lower in schizophrenia (*P* = 3.6 × 10^−7^, Kolmogorov-Smirnov two-sample test on the cumulative distribution functions; Fig. 1a) compared to genes with the lowest association. We did not observe a significant difference between the evolutionary rate of highly and lowly associated genes with coronary artery disease (*P* = 0.45, Kolmogorov-Smirnov two-sample test on the cumulative distribution functions; Fig. 1b). We also examined the correlation between the evolutionary rate of genes and their association with each disease. Genes that were highly associated with schizophrenia had a lower evolutionary rate (*R =* −0.07, *P* ∼10^−14^, Wilcoxon rank-sum test; supplementary fig. S1, Supplementary Material online), and this negative correlation stayed significant even after adjusting for the expression level (*R* = −0.03, *P* ∼10^−4^, Wilcoxon rank-sum test). For coronary artery disease, we did not find a significant correlation between their disease association and evolutionary rate (supplementary fig. S1, Supplementary Material online).

Because the associated genes with many complex traits show specific enrichment in different tissues (Ongen et al. 2017; Watanabe et al. 2019c), we compared the expression level of highly and lowly associated genes using the expression data of human genes in 54 distinct tissue types from the GTEx database (GTEx-Consortium 2020), quantified in the unit of the number of transcripts per million mapped reads (TPM) (supplementary table S2, Supplementary Material online). For schizophrenia, brain tissues were the tissues with the most significant difference in the expression of highly and lowly associated genes (*P* ∼10^−25^ in brain tissues compared to *P* ∼10^−10^ for the rest of tissues, Wilcoxon rank-sum test; Fig. 1c). For coronary artery disease, the cardiac, aortic/vascular, liver, and blood tissues showed the most significant difference in expression level of highly and lowly associated genes. This difference was the least significant in brain tissues (Fig. 1d). We systematically investigated the differences between the evolutionary rate and expression level of highly and lowly associated genes by repeating our comparisons across varying gene set sizes: 5,000, 500, and 50 genes. This approach allowed us to assess the impact of gene set size on our conclusions. We successfully replicated our findings for the cases of 5,000 and 500 genes; however, the results were not consistent for the 50-gene set, likely due to the small sample size (supplementary figs. S2 and S3, Supplementary Material online). Later in our analyses and across a broad spectrum of polygenic traits, we will utilize genome-wide correlations and implement statistical procedures to mitigate the inherent imbalance within datasets, ensuring robust and balanced inferences.

**Fig. 2.**
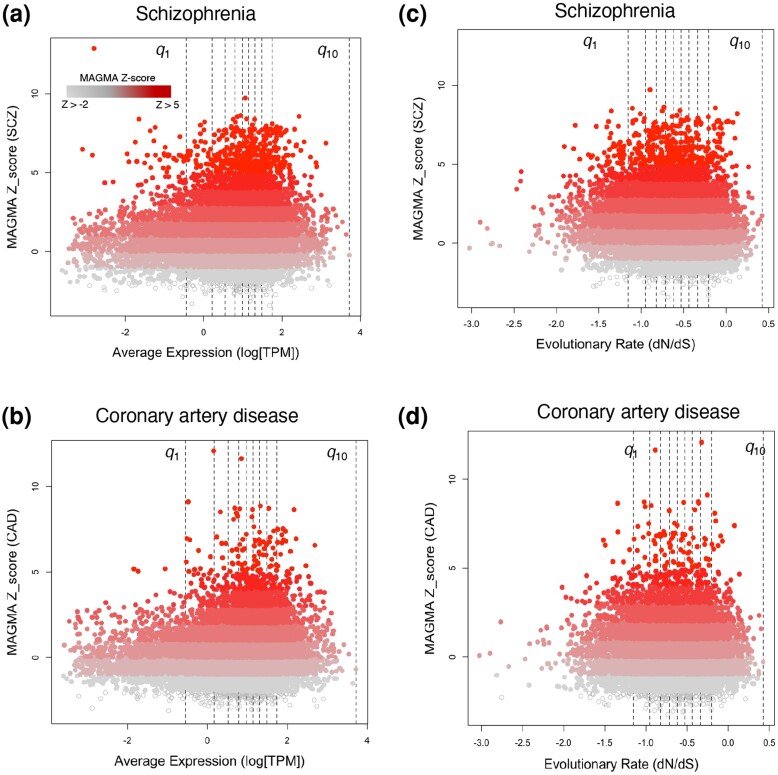
The expression and evolutionary rate quantiles of genes associated with schizophrenia and coronary artery disease. a, b) MAGMA *z-*scores of human genes versus their expression level (logarithm of the number of RNA transcript copies per million mapped reads) for schizophrenia (panel a), and coronary artery disease (panel b). c, d) MAGMA *z-*scores of human genes versus their evolutionary rate (logarithm of dN/dS) for schizophrenia (panel c), and coronary artery disease (panel d). The color scheme represents genes with MAGMA *z-*scores greater than different thresholds for their association with each disease, ranging from −2 (shown in gray) to 5 (shown in red). The vertical dashed lines in all panels represent deciles of the average expression level (panels a and b), and the evolutionary rate (panels c and d) of 14568 human genes.

Next, we explored how the expression level and evolutionary rate of genes associated with schizophrenia and coronary artery diseases vary as their degree of genetic association increases (Fig. 2). This involved comparing the gene expression levels and the evolutionary rates across various categories of genetic association (MAGMA *z-*scores) with those of human genes sorted into their corresponding deciles (supplementary fig. S4, Supplementary Material online, supplementary table S1, Supplementary Material online). In schizophrenia, the expression level of highly associated genes (MAGMA *z-*score > 5) was similar to the expression level of human genes within the sixth decile of expression and significantly different from any other decile (Wilcoxon rank-sum test, *P*_adj_ < 0.05; Bonferroni correction, supplementary fig. S4, Supplementary Material online, supplementary table S1, Supplementary Material online). For the coronary artery diseases, the expression level of genes with MAGMA *z-*scores > 5 was similar to the expression level of human genes within the sixth and seventh decile of expression but significantly different from any other decile (Wilcoxon rank-sum test, *P*_adj_ < 0.05; Bonferroni correction). In the case of evolutionary rate, the average evolutionary rate of highly associated genes to both diseases was comparable to that of genes within the fifth decile of expression level and significantly different from the evolutionary rate of human genes in other deciles (Wilcoxon rank-sum test, *P*_adj_ < 0.05; Bonferroni correction, For the full list of *P*-values see supplementary table S1, Supplementary Material online).

Overall, these results suggest that the interplay among genetic association, expression level, and evolutionary rate varies between schizophrenia and coronary artery disease. Genes highly associated with either schizophrenia or coronary artery disease tend to have a higher expression level compared to nonassociated genes. However, genes associated with schizophrenia exhibit a lower evolutionary rate compared to nonassociated genes, a pattern that was absent in the case of coronary artery disease. This indicates potential trait-specific relationships that differ across distinct traits. Importantly, this difference does not appear to be specific to any particular tissue, an aspect we will investigate later in this study. Before then, we address our second focal question: Do the evolutionary rates of genes associated with schizophrenia or coronary artery disease differ from genes implicated in monogenic diseases?

### Comparing the Evolutionary Rate of the Associated Genes With Schizophrenia or Coronary Artery Disease With the Genes Implicated in Monogenic Diseases

Our trait-specific approach allows us to compare an extensive set of genes associated with polygenic diseases with those implicated in monogenic diseases. This analysis is particularly important as complex and polygenic diseases have a more intricate etiology and are caused by multiple genes, compared to monogenic diseases. Previous studies have attempted this comparison but only with a small set of genes (Podder and Ghosh 2010). We compared the evolutionary rate of genes associated with schizophrenia and coronary artery disease with those implicated in monogenic diseases (*n* = 867) from the Disease Gene Conserved Sequence Tags (DG-CST) database (Boccia et al. 2005) and compiled by Podder et al. (Podder and Ghosh 2010) (supplementary table S3, Supplementary Material online).

**Fig. 3.**
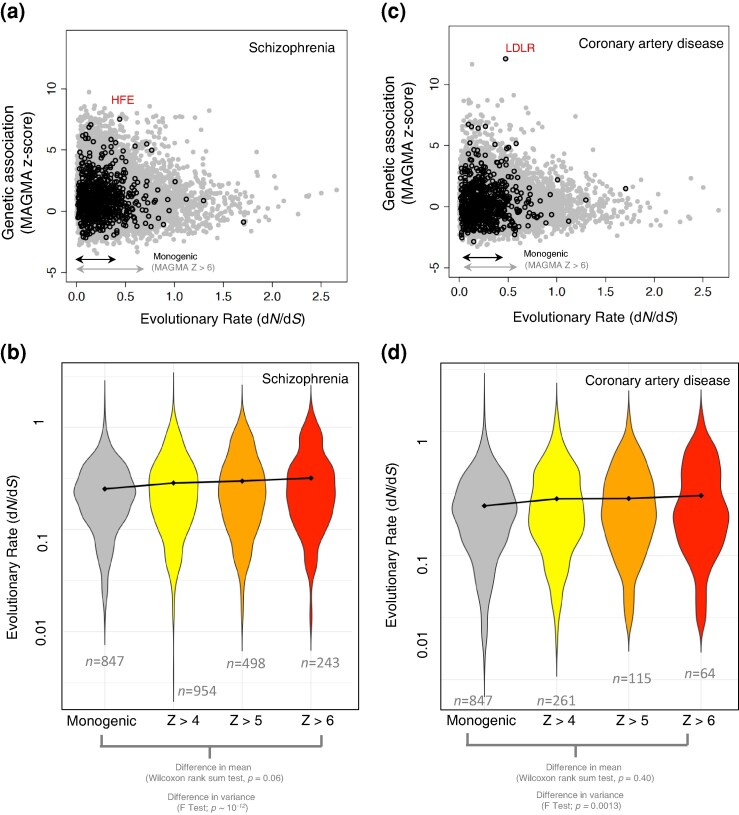
Evolutionary rates of genes implicated in monogenic and polygenic diseases. a) The MAGMA *z-*score versus the evolutionary rate of human genes for association with schizophrenia. b) The evolutionary rates of genes implicated to monogenic diseases (*n* = 847), and the genes associated with schizophrenia. c) The MAGMA *z-*score versus the evolutionary rate of human genes for association with coronary artery disease. d) The evolutionary rates of genes implicated to monogenic diseases (*n* = 847), and the genes associated with the coronary artery disease. The circles with black line in panels a and c correspond to the genes implicated in different monogenic diseases, compiled in the DG-CST database (Boccia et al. 2005). The arrows in panels a and c correspond to the range of the evolutionary rate from the lowest 10th percentile to the highest 10th percentile for genes implicated in monogenic diseases, as well as genes exhibiting MAGMA *z-*scores > 6 associated with either schizophrenia (panel a) or coronary artery disease (panel c). This range is ∼0.07 to 0.63 for genes associated with the coronary artery disease and ∼0.06 to 0.63 for genes associated with schizophrenia. The corresponding range for the evolutionary rate of monogenic diseases is ∼0.06 to 0.47.

We first examined the presence of genes implicated in monogenic diseases within the associated genes with schizophrenia or coronary artery disease (Figs. 3a-c). Notably, several genes implicated in monogenic diseases were among the highly associated genes surpassing the genome-wide significance (MAGMA *P*-value < 2.84 × 10^−6^, *z-*score > 4.5). The two genes that we highlighted in Fig. 3 are LDLR, encoding the low-density lipoprotein receptor protein, and HFE, expressing the human homeostatic iron regulator protein, which are highly associated with coronary artery disease and schizophrenia, respectively (Figs. 3c-d). The mutations in the LDLR gene account for ∼80% of monogenic cases of Familial hypercholesterolemia (Defesche et al. 2017). Mutations in the gene HFE are also implicated in Hemochromatosis which occurs when body builds up excessive amounts of iron leading to potential damage and dysfunction in vital organs such as the liver, heart and pancreas. Accumulation of iron in the brain exacerbates the decline in brain function, leading to cognitive and motor impairments in both neurodegenerative diseases and the natural ageing process (Kalpouzos et al. 2021; Lotan et al. 2023). This overlap is in line with previous findings that for many complex traits, associated genes are also implicated in similar Mendelian traits (Freund et al. 2018).

We proceeded to explore the difference in the evolutionary rate of genes associated with either of our polygenic diseases (with varying levels of association strength, MAGMA *z-*scores > 4, 5, and 6) and genes implicated in monogenic diseases. We did not find statistically significant differences (Figs. 3b-d, Wilcoxon rank-sum test) which suggests that the evolutionary rate of highly associated genes with either of our polygenic diseases does not significantly differ from genes implicated in monogenic diseases. We also conducted the comparison after excluding the genes implicated in monogenic diseases from the list of highly associated genes, and the results remained consistent (Wilcoxon rank-sum test, *P*-values > 0.05).

Despite the lack of a significant difference in the average evolutionary rate, highly associated genes with either of the two polygenic diseases displayed a significantly higher variance in the evolutionary rate when compared to genes implicated in monogenic diseases (*P* ∼10^−12^ for schizophrenia and *P* = 0.0013 for coronary artery disease, F test of variance). This higher variance resulted from the emergence of a bimodal distribution of evolutionary rate among highly associated genes with both diseases (see supplementary Supplementary Note S1, Supplementary Material online, and supplementary table S2, Supplementary Material online). The modes of this distribution correspond to genes that had evolved with a low evolutionary rate (d*N*/d*S* = 0.15 in schizophrenia and d*N*/d*S* = 0.14 in coronary artery disease) and a high evolutionary rate (d*N*/d*S* = 1.26 in schizophrenia and d*N*/d*S* = 1.19 in coronary artery disease), compared to genes implicated in monogenic diseases (average d*N*/d*S* = 0.25).

We also checked the difference in the evolutionary rate of nonassociated genes (MAGMA *P*-value > 2.84 × 10^−6^; *z-*score < 4.5) and the genes implicated in monogenic diseases and found that the evolutionary rate of nonassociated genes is significantly higher compared to the genes implicated in monogenic diseases (*P* = 1.1 × 10^−5^, Wilcoxon rank-sum test). In summary, these findings suggest that the evolutionary rate of highly associated genes with both polygenic diseases we studied here does not significantly differ from the evolutionary rate of genes implicated in monogenic diseases. However, they are more variable suggesting that genes associated with polygenic traits might have evolved under varying selection pressures compared to genes implicated in monogenic diseases.

### The Difference in the Evolutionary Rates and Expression Levels Between the Associated and nonassociated Genes With Polygenic Traits

Next, we decided to explore the relationship between genetic association and the evolutionary rate and expression level of genes across a broader range of polygenic phenotypes. To achieve this, we compared the evolutionary rate and the expression level of associated and nonassociated genes to all polygenic phenotypes in the GWAS ATLAS database (*n* = 4,756) (Watanabe et al. 2019a). To discern the direction of change in either the expression level or the evolutionary rate for each trait, we performed one-tailed analysis tests to compare the property of interest (evolutionary rate or expression level) between associated and nonassociated genes. We used the MAGMA *P*-value of 2.84 × 10^−6^ as the genome-wide significance threshold and identified 405 traits for whom 50 or more genes were associated. We then randomly selected the same number of genes as the number of associated genes from the genes with no association (MAGMA *P*-value > 2.84 × 10^−6^), 1,000 times, and compared the evolutionary rate and expression level of such nonassociated gene sets with those of associated genes (supplementary fig. S5, Supplementary Material online). We then sorted traits based on the number of significant comparisons using a Wilcoxon's rank-sum test (*P* < 0.05), and determined the enrichment of distinct categories of complex traits within the four possible groups in our analysis using a *χ*^2^ test. These groups corresponded to traits whose associated genes had a higher rate of evolution (group 1), a lower rate of evolution (group 2), a higher expression level (group 3), and a lower expression level (group 4), compared to nonassociated genes. For each enrichment analysis, we chose the top 100 traits with the highest number of significant comparisons in the property of interest as the foreground set and used the set of 405 traits with 50 or more associated genes as the background set.

In the first group, the most overrepresented domain among polygenic traits, showing a higher rate of evolution in associated genes compared to nonassociated genes, belonged to immunological traits (*P* = 1.4 × 10^−6^; *χ*^2^ test; supplementary fig. S5, Supplementary Material online). In the second group, for whom the associated genes have a lower rate of evolution, metabolic traits were overrepresented (*P* = 1.4 × 10^−6^; *χ*^2^ test; supplementary fig. S5, Supplementary Material online). Interestingly, both immunological and metabolic traits that were overrepresented in the first two categories, are the first and the second overrepresented traits in the third group, for whom the highly associated genes have a higher expression level compared to nonassociated genes (*P* = 0.043; *χ*^2^ test; supplementary fig. S5, Supplementary Material online). Lastly, and for top 100 traits whose associated genes have a lower expression level compared to nonassociated genes, psychiatric traits were overrepresented (*P* = 1.06 × 10^−5^; *χ*^2^ test; supplementary fig. S5, Supplementary Material online). These results show that the relationships between the genetic association and either the expression level or the evolutionary rate differs across different domains of polygenic traits.

### Genome-wide Correlations Between the Genetic Association, Expression Level and the Evolutionary Rates in Polygenic Traits

**Fig. 4.**
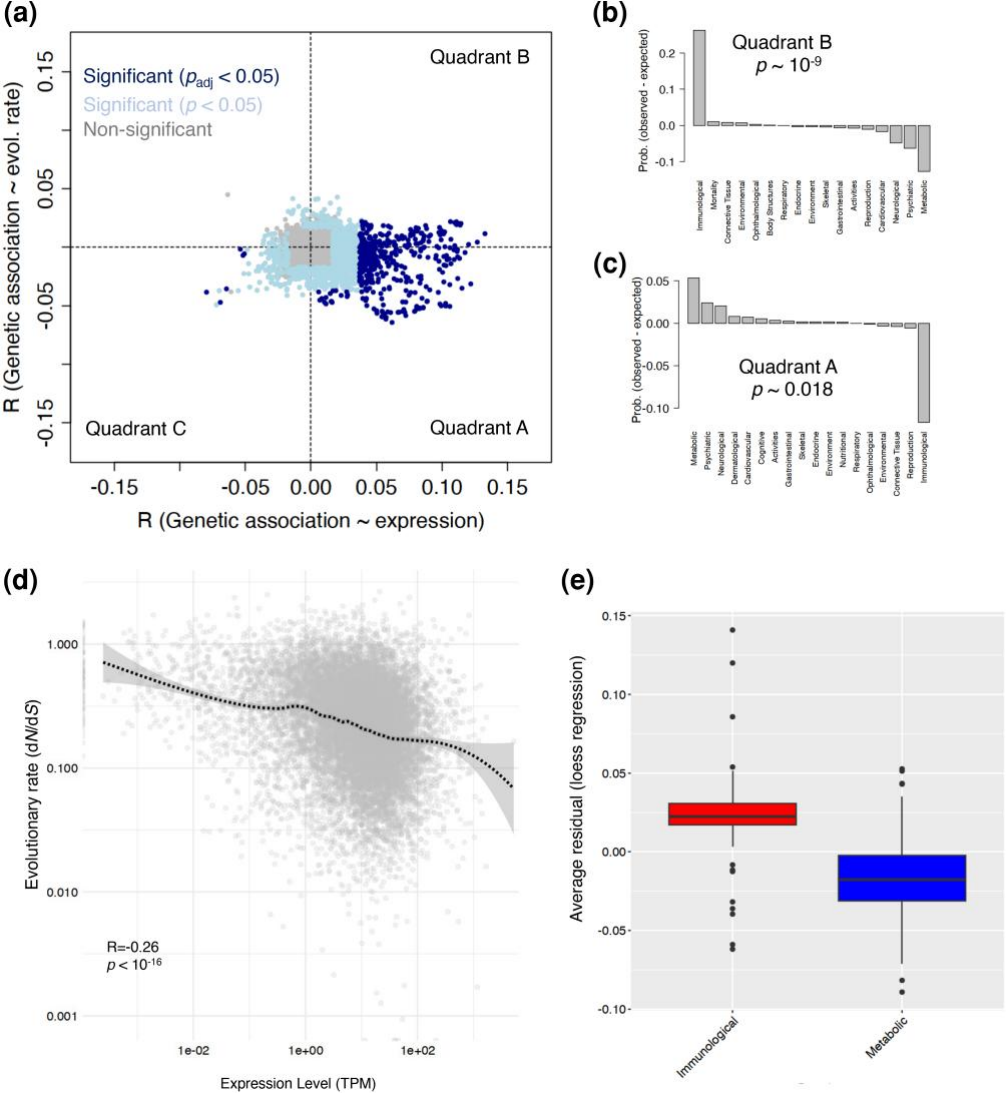
The relationship between genetic association and evolutionary rate and expression level varies in different categories of complex traits. a) The correlation between the negative logarithm of MAGMA *P*-value and evolutionary rate (*R*_rate_) versus the correlation between the negative logarithm of MAGMA *P*-value and expression level (*R*_expression_) for 4576 complex traits. The gray, light blue, and dark blue colors correspond to genes whose correlations (either *R*_rate_, or *R*_expression_) are nonsignificant, significant with a *P*-value < 0.05, and significant with a Bonferroni-corrected *P*-value (*P*_adj_) of 0.05, respectively. b, c) The difference in observed and expected fraction of different domains of polygenic traits in the quadrants B (panel b), and A (panel c). The *P*-values in these panels were calculated using a *χ*^2^ test. d) Evolutionary rate (d*N*/d*S*) versus the expression level of 15248 human genes in units of transcripts per million mapped reads with the loess line shown in dotted black. e) The average residual of genes associated with immunological traits (in red) and metabolic traits (in blue) from a loess regression between the evolutionary conservation and the expression level (data in panel d). In panel e, and for a reliable estimation of average values, we only considered traits with more than 50 associated genes (MAGMA *P*-values < 2.84 × 10^−6^).

To see whether the relationships between the genetic association and either the expression level or the evolutionary rate are apparent on a genome-wide scale we turned to our next analysis. For this purpose, we created a correlation diagram (Fig. 4a) and calculated the correlation between genetic association and the evolutionary rate (*R*_rate_, *y* axis) versus the correlation between genetic association and expression level (*R*_exp_, *x* axis) for all polygenic phenotypes in the GWAS ATLAS database. We particularly calculated partial correlations because of the known negative correlation between the expression level and the evolutionary rate of different genes caused by translational selection that is associated with high expression (Drummond and Wilke 2008). This will ensure an independent relationship between genetic association (–log_10_ [MAGMA *P*-value]) and either expression level or evolutionary rate. We also controlled for gene length as a covariate due to its substantial correlation with the genetic association of human genes across various polygenic traits, which we will discuss later in our work.

We found a significant correlation between genetic association and both the evolutionary rate (d*N*/d*S*) and the expression level for 1,657 traits (*P* < 0.05, Spearman's rank correlation). Upon correction for multiple comparisons (Bonferroni correction) the correlations remained significant for 436 traits. The majority of these polygenic traits (∼69%, 299 out of 436 traits) were in the quadrant A of the correlation diagram (Fig. 4a), showing a positive correlation between genetic association and expression level (*R*_exp_ > 0), but a negative correlation between genetic association and evolutionary rates (*R*_rate_ < 0) (Fig. 4a). For these traits, highly expressed genes are more strongly associated with the MAGMA *P*-values compared to lowly expressed genes. Interestingly, we found that metabolic traits were overrepresented among the traits in quadrant A (Fig. 4b; *P* = 0.0007, *χ*^2^ test). Several measures of fat to muscle ratio as well as body mass index were among the highly enriched traits within this quadrant. Since metabolic traits are closely related to the regulation of energy balance in the body, highly expressed genes associated with these traits may have a greater impact on metabolic processes and be under a stronger purifying selection (supplementary fig. S6, Supplementary Material online).

The second group of traits (quadrant B in Fig. 4a), for which the correlation between genetic association and both expression level and evolutionary rate was positive (∼30%, 131 of 436 traits) were enriched in immunological traits (Fig. 4b; *P* ∼10^−16^, *χ*^2^ test). This included the traits related to blood cell counts such as the counts of white blood cells, myeloid white cells, neutrophils and monocyte cells. Here, highly expressed genes were more strongly associated with polygenic phenotypes. Contrary to the expected negative correlation between the expression level and the evolutionary rate (Drummond et al. 2006; Drummond and Wilke 2008), these highly expressed and trait associated genes exhibited a higher rate of evolution compared to nonassociated genes. This unexpected higher rate of evolution suggests that positive selection may be acting on these genes, potentially due to their important role in the immune function.

We observed only six traits within the quadrant C of our correlation plot that passed the Bonferroni correction. These traits belonged to the domains of reproduction (three traits), body structure (two traits), and psychiatric traits (one trait). In these traits we observe a negative correlation between genetic association and both expression level and evolutionary rate (Fig. 4a). Genes that evolved at a lower rate were more associated with these traits compared to genes that evolved at a higher rate. Also, the highly associated genes to these traits had a lower expression level compared to lowly associated genes. Interestingly, we observed that the most highly associated gene with all six traits was *CSMD1* (MAGMA *P*-value < 10^−10^; supplementary fig. S7, Supplementary Material online). This gene encodes for the tumor suppressor protein “CUB and sushi domain-containing protein 1” that inhibits the myogenic sarcoma cell migration (Tang et al. 2012). The evolutionary rate (d*N*/d*S*) and the expression level of *CSMD1* are 0.1 and 0.69, respectively, positioning this gene within the lowest 10% of expression level and the lowest 15% of evolutionary rate among all human genes.

Our analysis did not reveal any polygenic phenotypes for which genetic association showed a significant negative correlation with expression level (*R*_exp_ < 0) and a positive correlation with evolutionary rate (*R*_rate_ > 0) (quadrant D in Fig. 4a). Although this pattern initially appears as an extension of the trend observed in quadrant A, where highly expressed genes tend to evolve slowly, we did not find any polygenic phenotype for which the associated genes had a lower expression level and a higher evolutionary rate compared to nonassociated genes. This observation suggests that lowly expressed and lowly conserved genes contribute less significantly to complex traits, compared to highly expressed and highly conserved genes. Indeed, as the expression level of human genes increases (and the evolutionary rate decreases) the number of significantly associated traits per gene (MAGMA *P*-value < 10^−7^) increases (*R* = 0.14, *P*∼10^−16^; Spearman's rank correlation, supplementary fig. S7, Supplementary Material online).

Although we conducted a partial correlation analysis, we aimed to determine the excess higher or lower purifying selection on different genes, mitigating potential bias stemming from the negative correlation between the expression level and the evolutionary rate of human genes (Fig. 4d) (Drummond and Wilke 2008). For instance, metabolic traits might display a lower correlation between genetic association and the evolutionary rate due to the bias introduced by the higher expression of genes associated with this trait. Similarly, immunological traits might exhibit a higher evolutionary rate due to their lower expression levels. We employed a locally estimated scatterplot smoothing (loess) regression between d*N*/d*S* and expression level and then aggregated the residuals of highly associated genes (MAGMA *P*-value < 2.8 × 10^−5^) for all 4,567 traits in the GWAS atlas. If immunological traits tend toward a higher evolutionary rate and metabolic traits tend toward a lower rate, we expect the average residual of genes associated with these traits to be positive and negative, respectively. This was indeed the case. As shown in Fig. 4e, immunological traits displayed a significantly positive average residuals (*P* < 0.001, t-test), indicating that genes associated with these traits showed higher evolutionary rates than expected when adjusted for the expression level. Conversely, metabolic traits exhibited a d*N*/d*S* ratio lower than anticipated when accounting for the expression level (*P* < 0.001, t-test).

In the last step of our evolutionary rate analysis, we aimed to get a more refined understanding of how natural selection has influenced the genetic variations within the genes associated with complex phenotypes. The metric for the evolutionary rate of the genes that we used in this study, d*N*/d*S*, provides an average estimation across the entire gene, thereby overlooking the variation in selective forces acting on different regions (Yang et al. 2005b; Yang and Dos Reis 2010; Bricout et al. 2023). To address this limitation, we calculated the proportion of sites within the coding regions of human genes that have evolved under purifying selection. We reasoned that this estimate is a more reliable indicator of purifying selection compared to the average and whole gene d*N*/d*S*, as the latter is susceptible to averaging biases. For instance, a minor fraction of sites with extreme low or high evolutionary rates can significantly influence the average values, potentially misleading the conclusions. We fitted different codon models to 20,362 human genes with available orthologs in chimpanzee (see Methods; supplementary table S5, Supplementary Material online). These models allowed us to quantify the strength of selection acting on these genes. Particularly and for this analysis, we calculated the fraction of codons with d*N*/d*S* < 1 to obtain, per each gene, the fraction of sites under purifying selection using the M8 model (see Methods, supplementary table S5, Supplementary Material online). Using this data, we ranked complex traits based on the average fraction of sites under purifying selection. Notably, metabolic traits were overrepresented among the top 100 traits whose associated genes have the highest fraction of sites under purifying selection (supplementary fig. S9, Supplementary Material online). We repeated this analysis for the top 200, and 300 traits whose associated genes have the highest fraction of sites under purifying selection (supplementary fig. S9, Supplementary Material online). In these analyses too, metabolic traits accounted for over 50% of all traits and were significantly overrepresented in our datasets (*P* ∼0.0005; *χ*^2^ test of enrichment). Altogether, these observations complement our enrichment analyses and illustrate the varying degree of purifying selection across distinct domains of polygenic traits. Notably, metabolic traits rank highest, indicating a greater likelihood of evolution under purifying selection. We will investigate the traits that potentially evolved under positive selection, utilizing polymorphism data later in this work. Before then we shift focus to explore the tissue-specific nature of relationships between genetic association and expression level in different traits.

### The Relationship Between Genetic Association and Expression Level Remains Consistent Across Different Tissues

We also examined how the tissue specificity of the correlations between genetic association and expression level might affect our findings. We reasoned that since our correlations were based on the average expression level of different genes, the presence of tissue-specific expression could potentially impact them. To systematically investigate this effect, we first identified genes associated with our traits of interest by ranking them according to their MAGMA *P*-values. We then selected the top 1,000 associated genes and performed TissueEnrich analysis (see Methods) to identify tissue-specific genes. Here, and for computational tractability, we restricted our analysis to 29 major tissues available in the GTEx database (supplementary table S2, Supplementary Material online), considering the entire human genome as the background reference.

**Fig. 5.**
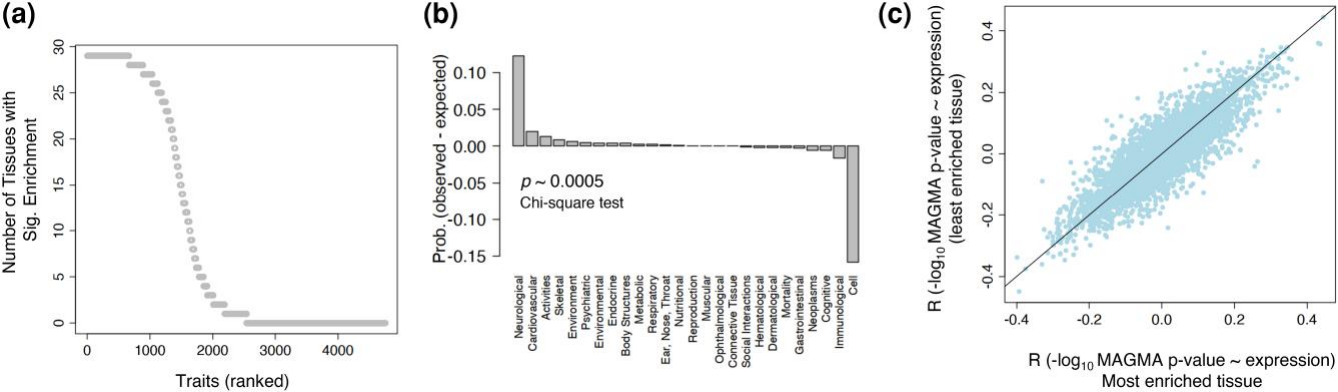
The relationship between genetic association and expression level does not significantly change by the substantial tissue specificity of genes associated with complex traits. a) The number of tissues with a significant enrichment of highly associated genes to each trait. b) The enrichment of different domains of complex traits within traits showing enrichment across all tissues (∼14% of all traits; 665 out of 4756 traits). c) The Spearman's correlation coefficient between the negative base-10 logarithm of MAGMA *P*-values of genes associated with complex traits and their expression level in the least enriched tissue (*y* axis) plotted against the same correlation in the most enriched tissue (*x* axis).

Our results revealed a spectrum of traits for whom the highly associated genes were enriched in different tissues (Fig. 5a; supplementary table S4, Supplementary Material online). The extreme cases corresponded to traits that lacked enrichment in any specific tissue (∼47% of all traits; 2,215 out of 4,756 traits), and traits showing enrichment across all tissues (∼14% of all traits; 665 out of 4,756 traits). No specific domain of complex traits was over- or underrepresented among the traits without specific tissue enrichment. However, neurological and cardiovascular traits were overrepresented among the traits that showed enrichment across all tissues (*P* ∼0.0005; *χ*^2^ test).

We then investigated whether tissue specificity can change the strength of correlation between genetic association and the expression level. To do so and for each trait, we performed two comparisons. First, we calculated the correlation between genetic association and expression level in the tissue that showed the highest enrichment of associated genes and compared it with the average gene expression across all tissues (supplementary table S4, Supplementary Material online). Second, we compared the correlation between genetic association and expression level in the tissues with the highest and lowest enrichment of 1,000 associated genes to each polygenic trait. In both cases, we used Fisher *z-*transformation to see whether the correlation strength significantly changes from one tissue to another (see Methods). Remarkably, and in both comparisons, we found no significant changes in the correlation between genetic association and expression level in different tissues. We also repeated our enrichment analysis with only 50 highly associated genes with each trait to see how a smaller gene set might affect our result. In this analysis, too, we did not find tissue specificity to significantly change the correlation between genetic association and the expression level of associated genes. We conclude that although for many traits, the associated genes are specifically expressed in several tissues, the overall relationship between genetic association and expression level is consistent across different tissues. This further extends our previous observations in schizophrenia and coronary artery disease that the difference in the expression of highly and lowly associated genes remains significant in all tissues.

### Short-term Evolution of Genes Associated With Polygenic Traits

Our d*N*/d*S* metric relies primarily on the interspecies divergence data, making it less sensitive to identify the patterns of selection at lower levels of divergence and over short evolutionary timescales (Ho et al. 2005; Kryazhimskiy and Plotkin 2008). In complex traits, short-term evolutionary dynamic plays an important role in shaping genetic diversity within populations such as rapid adaptation or response to environmental pressures (Jain and Stephan 2017; Barghi et al. 2020). To better account for this factor, we used the polymorphism data to estimate the direction of selection (DoS) (Stoletzki and Eyre-Walker 2011; Moutinho et al. 2019a, 2019b). In brief, DoS captures the deviation from the assumption of strict neutrality using the number of synonymous substitutions (*D_s_*), the number of nonsynonymous substitutions (*D_n_*), the number of synonymous polymorphism (*P_s_*), and the number of nonsynonymous polymorphism (*P_n_*):


(1) $$\mathrm{Direction}\;\mathrm{of}\;\mathrm{selection}\;=\frac{D_{n}}{D_{n}+D_{s}}-\frac{P_{n}}{P_{n}+P_{s}}\;$$

**Fig. 6.**
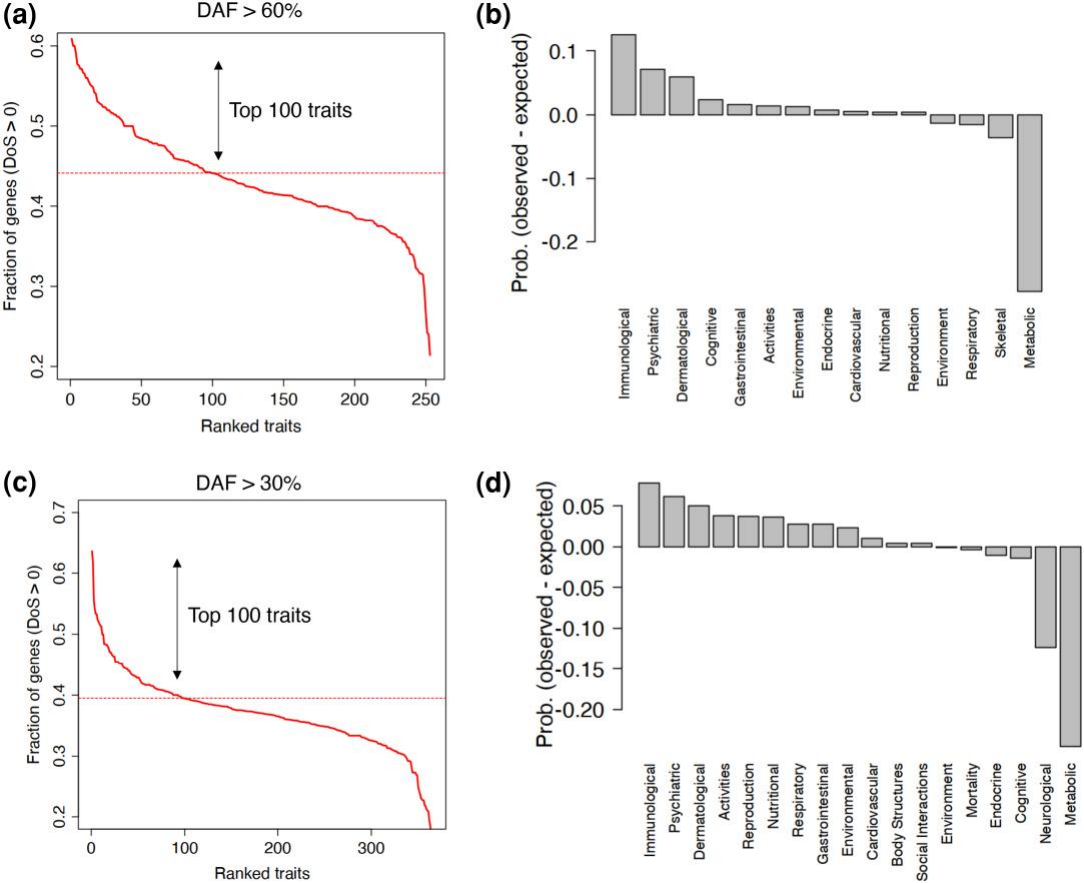
Genes associated with immunological traits have a high fraction of positive direction of selection. a) The ranked fraction of associated genes (MAGMA *P*-value < 2.8 × 10^−5^) with a positive direction of selection (Equation 1) in different polygenic traits having variants with a derived allele frequency > 60%. b) The difference in the observed and expected fraction of different domains of polygenic traits within 100 traits with the highest fraction of positive degree of selection. c) The ranked fraction of associated genes (MAGMA *P*-value < 2.8 × 10^−5^) with a positive direction of selection (Equation 1) in different polygenic traits having variants with a derived allele frequency > 30%. d) The difference in observed and expected fraction of different domains of polygenic traits within 100 traits with the highest fraction of positive degree of selection. The enrichments in panels b and d were significant with *P*-values < 0.005 using a *χ*^2^ test of enrichment.

The negative or positive values of DoS indicate that the gene of interest is more likely evolving under purifying or positive selection, respectively. We calculated the average DoS for each human complex trait for whom highly associated genes had the derived allele frequencies more than 60%. We applied this threshold to ensure that the accumulation of slightly deleterious mutations would not bias our estimations (see Methods). We then calculated the enrichment of different domains of polygenic traits among the top 100 traits exhibiting the highest proportion of genes with a positive direction of selection. Consistent with previous analyses, immunological traits were notably overrepresented (∼30% of all traits, *P* ∼0.0005; *χ*^2^ test of enrichment), (Fig. 6b). We obtained the same results when used the minimum derived allele frequency of 30% (Figs. 6c-d). Of note, the choice of derived allele frequency affects the feasibility of this analysis such that a high derived allele frequency favors genes with positive degree of selection and a low derived allele frequency favors genes with negative degree of selection. For example, with a derived allele frequency > 60%, only 3 traits met the criteria of having more than 50% of genes with a negative DoS. When we relaxed this threshold to 1%, the number of traits with a negative degree of selection increased to 156, yet none exhibited 50% or more of their associated genes with a positive DoS. We thus conducted this analysis with relatively stringent thresholds for derived allele frequency (30% and 60%) to minimize the impact of slightly deleterious mutations.

### Biases in the GWAS ATLAS that Can Influence Genome-wide Observations

Our conclusions in this work could be affected by the presence of different biases that exist in the GWAS atlas. Here, we focus on three sources of bias: a gene length bias as the association of human genes with different polygenic traits might be influenced by the length of genes, an ascertainment bias because the same trait has been studied with different genome-wide associate studies, and a power bias as the GWAS atlas contains GWASs with different powers.

We have systematically addressed the gene length bias in our analyses of the correlations between genetic association and either the evolutionary rate or the expression level of distinct human genes (Fig. 4). The significance of gene length extends beyond the simple notion that longer genes tend to encompass more SNPs within a GWAS dataset, thereby increasing the likelihood of significant markers. Longer genes possess a higher propensity for diverse functionalities, exhibit increased co-expression, and engage in a higher number of protein-protein interactions, compared to shorter genes (Lopes et al. 2021). Notably, gene length demonstrates a weak correlation with both d*N*/d*S* (Spearman's *R* = −0.08, *P* ∼10^−16^) and expression level (Spearman's *R* = −0.07, *P* ∼10^−16^). However, it exerts a considerable bias on MAGMA *P*-values across various traits, reaching as high as Spearman's *R* = 0.6 (*P* ∼10^−16^). Our observations show that psychiatric traits are notably influenced the most by this bias (supplementary fig. S10, Supplementary Material online).

The second bias, known as the ascertainment bias, arises due to the existence of various GWASs investigating the same trait (e.g. schizophrenia). In our enrichment analyses, both the foreground and background sets encompass traits investigated across various GWASs, which likely mitigates the impact of ascertainment bias. To assess this bias more quantitatively, we calculated the correlation between the number of different traits within each domain and chapter of polygenic traits and the correlation between MAGMA *z-*scores and gene expression levels and evolutionary rates (*R*_exp_ and *R*_rate_). We did not find the number of traits to significantly correlate with our genome-wide correlations (supplementary fig. S11, Supplementary Material online). This shows that the number of distinct GWASs investigating a trait has minimal influence on the genome-wide correlations between genetic association and either evolutionary rate or expression level.

The third bias we explored was the GWAS power, wherein a larger participant pool often leads to the identification of more causal genes associated with specific traits. Indeed, polygenic traits that were studied using more powerful GWASs, on average, demonstrated a higher correlation between genetic association and expression level (*R* = 0.53, *P* < 10^−16^; Spearman's rank correlation) and a lower correlation between genetic association and evolutionary conservation (*R* = −0.21, *P* < 10^−16^; Spearman's rank correlation), compared to GWASs with a lower power. To see which domains of polygenic traits exhibited a significantly higher *R*_exp_ and *R*_rate_ independent of GWAS power, we employed a loess regression and explored the enrichment of different trait domains while accounting for the GWAS power. Specifically, we investigated two groups of traits in line with our correlational analysis: (i) traits displaying high *R*_exp_ and high *R*_rate_, and (ii) traits with high *R*_exp_ and low *R*_rate_ (supplementary fig. S12, Supplementary Material online). Immunological traits were indeed enriched among traits demonstrating high *R*_exp_ and high *R*_rate_ after adjusting for GWAS power (*P* = 0.00049, *χ*^2^ test). Metabolic traits emerged as the second domain enriched in traits exhibiting high *R*_rate_ and low *R*_exp_, with neurological traits constituting the first domain (*P* = 0.0001, *χ*^2^ test). These findings reinforce our main conclusions regarding the enrichment of metabolic and immunological traits and further underscore how GWAS power can influence the correlation between genetic association and both expression level and evolutionary rate.

## Discussion

Our study reveals several features for the relationship between expression level, evolutionary rate, and genetic association of human genes to complex phenotypes. Focusing on schizophrenia and coronary artery disease as two polygenic phenotypes with a low genetic correlation, we investigated how the evolutionary rate and expression level of associated genes differ from those not associated with these polygenic diseases. We found that highly associated genes exhibited a significantly higher expression level compared to nonassociated genes in both diseases. Notably, in both diseases, genes displaying extremely high or low expression levels (and evolutionary rates) did not significantly contribute to the genetic association. This finding potentially reinforces the concept of intermediate essentiality for disease-relevant genes in humans (Jain and Stephan 2017). The functional importance of these genes lies between the extremes of genes with low essentiality that tolerate genetic changes and genes with high essentiality for whom most changes are lethal (Stoletzki and Eyre-Walker 2011; Barghi et al. 2020).

By investigating 4,576 complex traits in the GWAS ATLAS database, we explored the relationship between genetic correlation, gene expression level, evolutionary rate, and estimates of selection pressure in human genes across a broader spectrum of polygenic traits. Our findings reveal a diverse relationship between the association of various genes with complex phenotypes and their corresponding expression level and evolutionary rates. Particularly, two groups of polygenic traits were distinguished. The first group consisted of metabolic traits. The genes associated with metabolic traits exhibited a high expression level, and a low evolutionary rate. They further have the highest proportion of sites evolving under purifying selection. These results extend previous observations that both the function and topology of central metabolism have evolved under purifying selection over long evolutionary times (Beaumont 1988; Petit and Barbadilla 2009; Maddamsetti 2022). The second group of complex traits corresponded to immunological traits for whom highly associated genes had a high expression level and a high evolutionary rate that is likely indicative of positive selection in these genes. Indeed, proteins involved in the immune response are preferential targets of positive selection in many species, including mammals (Kosiol et al. 2008) and specifically primates (Bustamante et al. 2005; Arbiza et al. 2006; Gibbs et al. 2007; Barreiro and Quintana-Murci 2010). It is crucial to emphasize the significant heterogeneity in the genome-wide correlations we studied here across domains of polygenic traits. Even within the same domain, we observe varied correlations between genetic association and either expression level or evolutionary rate among different traits. Our work demonstrates enrichments within specific domains, suggesting tendencies rather than universally applicable characteristics for all traits within a particular domain of polygenic traits.

The correlations observed between genetic association, evolutionary rate, and expression level in polygenic traits tend to exhibit low effect sizes, a common occurrence in such traits. For instance, studies utilizing gene-based association with reference transcriptomic data have revealed a low heritability estimate for gene expression, with SNP-h^2^ below 0.15 across various prediction models (Gamazon et al. 2015). This modest heritability is largely attributed to environmental influences like lifestyle and diet, which significantly impact gene expression variability. Similarly, the evolutionary rate of genes associated with polygenic traits is recognized to undergo subtle changes, with polygenic adaptation often entailing minor shifts in allele frequencies and leaving minimal trace on genomic loci (Pritchard et al. 2010; Rosenberg et al. 2019; Barghi et al. 2020). These factors likely contribute to the relatively weak genome-wide correlations observed between genetic association and either expression level or evolutionary rate. Moreover, it is crucial to clarify that our study's objective was not to assess the reliability of evolutionary rate or expression level as predictors of genetic association. Instead, our focus was on exploring potential differences in evolutionary processes acting on polygenic traits and utilizing these proxies to gain insights into such dynamics. Our findings reveal that while genome-wide correlations may be modest, they exhibit distinct tendencies across various domains of polygenic traits, with notable extremes observed in metabolic and immunological traits.

Our results contribute to resolving the disparities in comparing the evolutionary rates between genes associated with polygenic traits and those not associated. For most polygenic traits we observed a positive correlation between the expression level and genetic association. In approximately 69% of polygenic traits, the associated genes have a lower evolutionary rate compared to nonassociated genes. Conversely, in around 30% of these traits, we observed a higher evolutionary rate in the associated genes compared to the nonassociated ones. We further corroborated this observation by showing that genes with a higher expression level and a lower evolutionary rate are generally more pleiotropic and associated with more traits compared to the genes with a lower expression level and a higher evolutionary rate (supplementary fig. S8, Supplementary Material online). We also observed the lack of a significant negative correlation between genetic association and expression level, and a positive correlation between genetic association and the evolutionary rate of different genes. It is important to clarify that this does not imply that lowly expressed and fast-evolving genes do not contribute to complex traits. On the contrary, various traits are enriched in both categories of genes (supplementary fig. S13-S14, Supplementary Material online). What this suggests is that as the association of a gene with a complex trait increases, it appears less likely for this gene to have a lower expression level and a higher evolutionary rate compared to nonassociated genes. One plausible explanation could be that complex traits are manifested through an intricate network of gene-gene and protein-protein interactions (Barrio-Hernandez et al. 2023). Lowly expressed and fast-evolving genes might be less likely to interact with other genes (Hahn and Kern 2005; Lemos et al. 2005; Drummond et al. 2006; Drummond and Wilke 2008), potentially leading to reduced importance in complex trait manifestation.

Our findings are also relevant for understanding polygenic adaptation, a process involving multiple genetic variants undergoing natural selection, collectively contributing to the population's adaptation to its environment. Notably, in about 30% of polygenic traits, associated genes have a higher rate of evolution compared to nonassociated genes, with several traits showing more than 50% of associated genes favoring positive selection (Fig. 6). This accelerated evolutionary rate potentially indicates signs of polygenic adaptation. Exploring the prevalence of such adaptive processes in contemporary human populations using methodologies like the singleton density score (Field et al. 2016) remains an interesting avenue for future investigation. An analysis of the overlap between these approaches can potentially disentangle which human polygenic phenotypes demonstrate long-term adaptive signals versus those more specific to certain populations.

In this study, we have undertaken a comprehensive analysis, considering genetic association, gene expression, and evolutionary rate, to explore the genome-wide signal for polygenic phenotypes. Notably, the integration of these three components can also be used for gene prioritization of GWAS significant loci in gene-dense regions. The majority of existing analyses have largely focused on integrating GWAS signals with gene expression regulation (e.g. through transcriptome-wide association studies, TWAS) without considering the evolutionary rate of genes. However, gene expression can exhibit correlations at the locus level due to linkage disequilibrium (LD) across expression quantitative trait loci (i.e. eQTL), as observed in TWAS analyses (i.e. the different genes in a locus typically show similar associations). By incorporating evolutionary conservation into the association analysis it can be possible to disentangle the genetic signal more effectively by considering the potentially heterogeneous evolutionary rates of genes within a locus. In particular, genes with a higher evolutionary rate are more likely to be relevant for the analyzed trait and thus such an integrative analysis could be used for gene prioritization of GWAS significant loci considering both gene expression regulation and evolutionary rate.

It is important to acknowledge two key limitations of our study. Firstly, the majority of traits in the GWAS ATLAS database are primarily studied in European populations, potentially limiting the generalizability of our findings. Exploring these traits across diverse ethnicities while considering variations in LD structure, which affects allele frequencies (Cheng et al. 2022), the discovery rate of GWAS hits (Mostafavi et al. 2023), evolutionary rates (Wu and Zhang 2011), and expression levels (Gay et al. 2020), presents an intriguing avenue for future research. Cross-ancestry validations could provide insights into whether the architecture of polygenic traits is adaptable across different human populations (Wang et al. 2020). Secondly, our study relied on data from the third release of GWAS ATLAS (as of November 15, 2019) (Watanabe et al. 2019a), which might not include the most recent GWAS summary statistics. While we chose this dataset due to its comprehensive coverage of polygenic traits with standardized gene-based scoring (de Leeuw et al. 2015), it is crucial to replicate our findings in future studies using updated summary statistics from well-powered studies of biobank-scale datasets.

In conclusion, our study unravels the intricate relationship between genetic association, expression level, and evolutionary rate, revealing trait-specific variations. Further exploration of the causes of such variations will be essential for a comprehensive understanding of the genetic architecture underlying complex traits and diseases. We further established the polygenic evolution portal (evopolygen.de) as a resource for investigating relationships and generating hypotheses in the field of polygenic human trait evolution.

## Materials and Methods

### MAGMA Analyses of Coronary Artery Disease (CAD) and Schizophrenia (SCZ)

We applied the gene-analysis module from the MAGMA tool to identify gene-association for coronary artery disease and schizophrenia (de Leeuw et al. 2015). MAGMA computes *P*-values per gene by aggregating SNP-level association statistics within or near each gene to create a gene-level test statistic. An empirical null distribution is generated through permutations or simulations to represent the expected distribution of the test statistic under the null hypothesis. The *P*-value for each gene is then calculated as the proportion of null distribution values that are equal to or higher than the observed test statistic, indicating the gene's statistical significance in association with the trait or disease of interest. In order to account for correlations between nearby genetic variants, MAGMA incorporates LD structure of the regions. In our analysis, we accounted for LD structure using the European LD matrix from Phase 3 of 1,000 Genomes as provide by MAGMA (https://ctg.cncr.nl/software/magma). In order to compute gene-based association for CAD the genome-wide association study (GWAS) summary statistics from the Cardiogram consortium were used (available in: https://www.med.unc.edu/pgc/results-and-downloads) (Nikay et al. 2015). Concerning schizophrenia the gene-based association based on associations from the last available GWAS from the Psychiatric Genomic Consortium (PGC) were considered (available in: http://www.cardiogramplusc4d.org/media/cardiogramplusc4d-consortium/data-downloads/cad.additive.Oct2015.pub.zip) (Trubetskoy et al. 2022). Data are available through the Functional Mapping and Annotation (FUMA) GWAS platform (Watanabe et al. 2017b). We associated SNPs with genes within a 1.5-kilobase range on both sides and further compared the MAGMA *z-*scores using different window sizes of 0 bp, 500 bp, 1k bp, 1.5 kbp, 2k bp, and 50 kb to ensure the robustness of our approach. The MAGMA *z-*scores calculated using these window sizes were highly correlated (Spearman's *R* > 0.8, *P* < 10^−16^; supplementary fig. S15, Supplementary Material online).

We further calculated the genetic association using another common gene-based approach, VEGAS (Versatile Gene-based Association Study) (Liu et al. 2010). VEGAS uses a permutation-based framework, aggregating SNP-level association signals within genes, while MAGMA employs a methodology reliant on LD information from reference panels for gene analysis. Approximately 88% to 89% of genes displayed a nominal *P*-value of association (0.05) with VEGAS overlapped with the MAGMA results, indicating a substantial alignment between the two methods (see supplementary fig. S16, Supplementary Material online). Notably, both the direction and strength of correlations (*R*_exp_ and *R*_rate_) remained consistent using the gene-based statistic of VEGAS. Particularly, *R*_exp_ shifted from 0.08 (*P* < 10^−16^) using MAGMA to 0.06 (*P* ∼10^−11^) with VEGAS, and *R*_rate_ changed from −0.08 (*P* < 10^–16^) using MAGMA to −0.07 (*P* < 10^16^) with VEGAS.

### Genetic Correlation Between CAD and SCZ

To investigate the genetic correlation between coronary artery disease and schizophrenia, we employed LD score regression which leverages genome-wide summary statistics from large-scale GWAS to estimate the extent to which the genetic variants underlying one trait contribute to the variance of the other trait (Bulik-Sullivan et al. 2015). This approach employs regression analysis to examine the relationship between LD scores (i.e. sum of LD r^2^ measured considering all the variants in a given locus) and the test statistics of the single-nucleotide polymorphisms (SNPs) from the GWAS.

### Evolutionary Rate Calculations, and the Estimation of the Fraction of Sites Under Purifying Selection

We used the following datasets in our analyses. First, and for the correlation between evolutionary rate and genetic association of different genes (Fig. 4), we used the dataset of Chakraborty et al^3^. In this dataset, the average ratio of nonsynonymous substitutions per nonsynonymous site (d*N*) to synonymous substitutions per synonymous site (d*S*) is used as a metric for assessing the evolutionary rate of distinct genes. To ensure robustness, genes with a minimum of three orthologous pairs across the following species were considered: Macaque (*Macaca mulatta*), Gorilla (*Gorilla gorilla*), Orangutan (*Pongo abelii*), Chimpanzee (*Pan troglodytes*), and Gibbon (*Nomascus leucogenys*). The d*N*/d*S* values are then computed using the PAML suite (Yang 2007), and the average pairwise values of d*N*/d*S* for each human gene (total of 15,248) is calculated. Second, and for the site-specific analysis of the evolutionary rate of human genes (Fig. 6), we fitted the codon models M1, M2, M3, M7 and M8 (Yang et al. 2000a) in Codeml within the PAML suite to a pair of orthologous sequences of human and chimpanzee using the pairwise alignment of 20,362 genes that were previously compiled by Nielsen et al (Nielsen et al. 2005). We particularly used model M8 and summed the fraction of sites with d*N*/d*S* < 1 serving as an indicator of the degree to which a gene has undergone purifying selection. Third, and for the polymorphism data, we used the estimated degree of selection for human genes from Gaya-Vida et al (Gayà-Vidal and Albà 2014). This dataset contains human SNP data from the coding sequence of 9,785 genes with available rate of polymorphic nonsynonymous (*P_n_*), and synonymous (*P_s_*) mutations. We used the genes for whom the derive allele frequency was > 60% to minimize the bias that slightly deleterious mutations would have on the rate of evolution. We also relaxed this threshold to 1% and calculated the enrichment of different domains of polygenic traits within 100 traits with the most positive direction of selection.

### GWAS ATLAS Dataset and Expression Data

We used the third release of the GWAS ATLAS dataset (2019-11-15) with 4,756 GWAS summary statistics as well as MAGMA gene *P*-values. We used the Ensembl GRCh37 gene annotations to obtain the gene lengths used as a covariate in our correlational analyses. In the analysis of the fraction of purifying selection and the direction of selection we only considered genes whose MAGMA *P*-values were less than 2.84 × 10^−6^ as the significance threshold to control for multiple testing. We used the GTEx expression dataset (version 8) that encompasses the expression data of 54 tissues, sub-tissues and cell types (-Consortium 2020). We used the whole dataset for the analysis of the expression specificity of genes associated with schizophrenia and the coronary artery diseases. For the analysis of all traits within the GWAS atlas, we used the average expression in 29 main tissues without cell types. We excluded blood because of the significant heterogeneity of the gene expression of different cell types (-Consortium 2020).

### Trait and Tissue Enrichment Analysis

To analyze tissue specificity across all 4,756 GWAS in the GWAS ATLAS, we implemented a two-step process. First, we identified trait-specific genes by ranking all genes according to their MAGMA *P*-values. We considered the 1,000 most significant genes as input for the enrichment analysis using the TissueEnrich R package (Jain and Tuteja 2019). Tissue-specific genes were determined using the GTEx expression data (supplementary table S2, Supplementary Material online) (-Consortium 2020) and enrichment was performed using the hypergeometric test with the entire gene set as background.

We calculate the enrichment of polygenic traits in different quadrants of our correlation plot using the *χ*^2^ test. In brief, we constructed two sets of traits. The background set was the set of traits for whom the correlation between genetic association and either expression level (*R*_exp_) or evolutionary rate (*R*_rate_) was significant. The foreground set in each quadrant was the list of selected traits (e.g. 100, 200, or 300 traits) with the highest value of *R*_exp_*×R*_rate_. We counted the domains of polygenic traits and compared the expected proportions of such domains with the proportions observed in the foreground set. We then applied the *χ*^2^ test using the observed counts of traits within the foreground set and the expected proportions in the background test and reported the *P*-values from the test result.

### Statistical Analysis and Data Availability

To test the null hypothesis that the correlations between genetic association (MAGMA) and expression level are tissue-independent, we used Fisher's *z-*transformation. In this approach, Spearman's correlation coefficients are converted to *z-*scores, so that they become normally distributed. The null hypothesis is then tested using a t-test on the *z-*scores. All statistical analyses were performed using R (v4.2.2). We employed the “pcor.test” function from the “ppcor” package in R to control for the influence of the covariates, expression level and the evolutionary rate, while assessing the relationship between the genetic association function and each of these variables. The datasets and the scripts are available at the GitHub page: https://github.com/dasmeh/Complex_Trait_Evolution

## Supplementary Material

msae115_Supplementary_Data
