# Supplementary material for: Analysis of Evolutionary Conservation, Expression Level, and Genetic Association at a Genome-wide Scale Reveals Heterogeneity Across Polygenic Phenotypes
Source: Mol Biol Evol. 2024 Jun 12;41(7):msae115. doi: 10.1093/molbev/msae115 (PMC11247350; doi:10.1093/molbev/msae115)
Supplement: msae115_Supplementary_Data [file msae115_supplementary_data.zip › EvoPolyGen_SI_R3.pdf]

Supplementary Information

Analysis of Evolutionary Conservation, Expression Level and Genetic Association at a Genome-wide Scale Reveals Heterogeneity in Traits Across Polygenic Phenotypes

Ann-Sophie Giel[a], Jessica Bigge[a], Johannes Schumacher[1], Carlo Maj[1*], Pouria Dasmeh[1,2,3*]

[1]Centre for Human Genetics, Marburg University, Marburg, Germany. [2]Department of Chemistry and Chemical Biology, Harvard University, USA. [3]Institute for Evolutionary Biology and Environmental Studies, University of Zurich.

[a]Equal contributions, *Corresponding Authors (dasmeh@staff.uni-marburg.de; carlo.maj@uni-marburg.de)

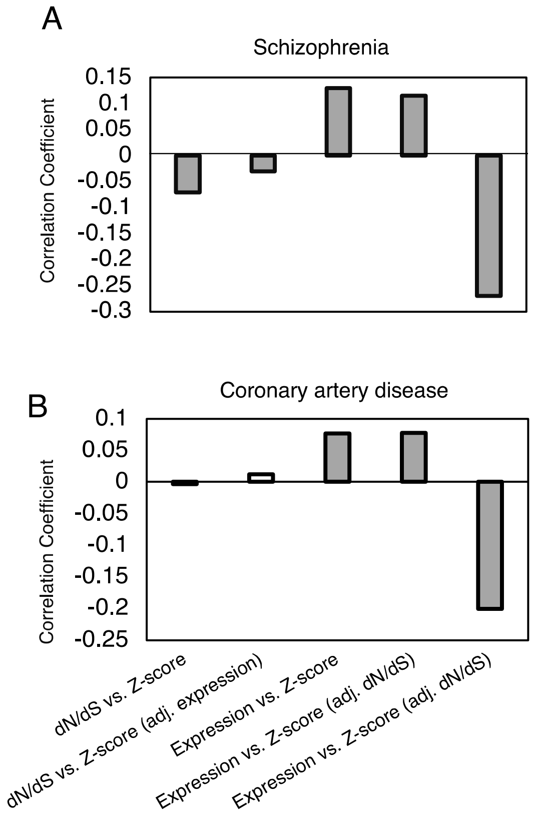

Figure S1. The Spearman correlation coefficient of MAGMA z-scores for ~ 18,000 genes with their evolutionary rate (dN/dS), and expression levels for A) schizophrenia, and B) coronary artery disease. We performed partial correlation analyses to assess the correlation between MAGMA z-scores and various features (such as evolutionary rate), while controlling for the confounding effect of the second variable (i.e., expression level).

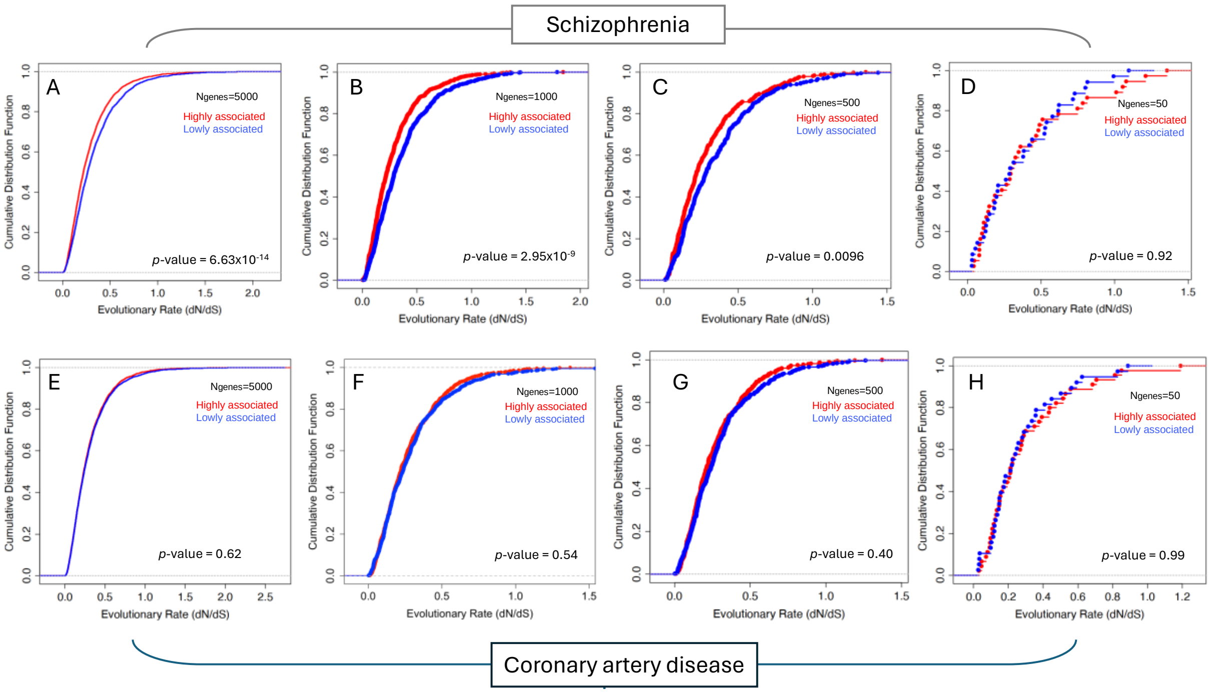

Figure S2. The difference between the distributions of the evolutionary rate of highly and lowly associated genes to schizophrenia (panels A-D), and the coronary artery disease (panels E to H). In panels A-D (schizophrenia), and panels E-H (coronary artery disease), we compared 5000, 1000, 500 and 50 highly associated genes (shown in red) with the same number of lowly associated genes (shown in blue). We used MAGMA z-scores to select these genes such that highly and lowly associated genes have the highest and the lowest z-scores. The p-values in all panels were calculated using the Kolmogorov-Smirnov test.

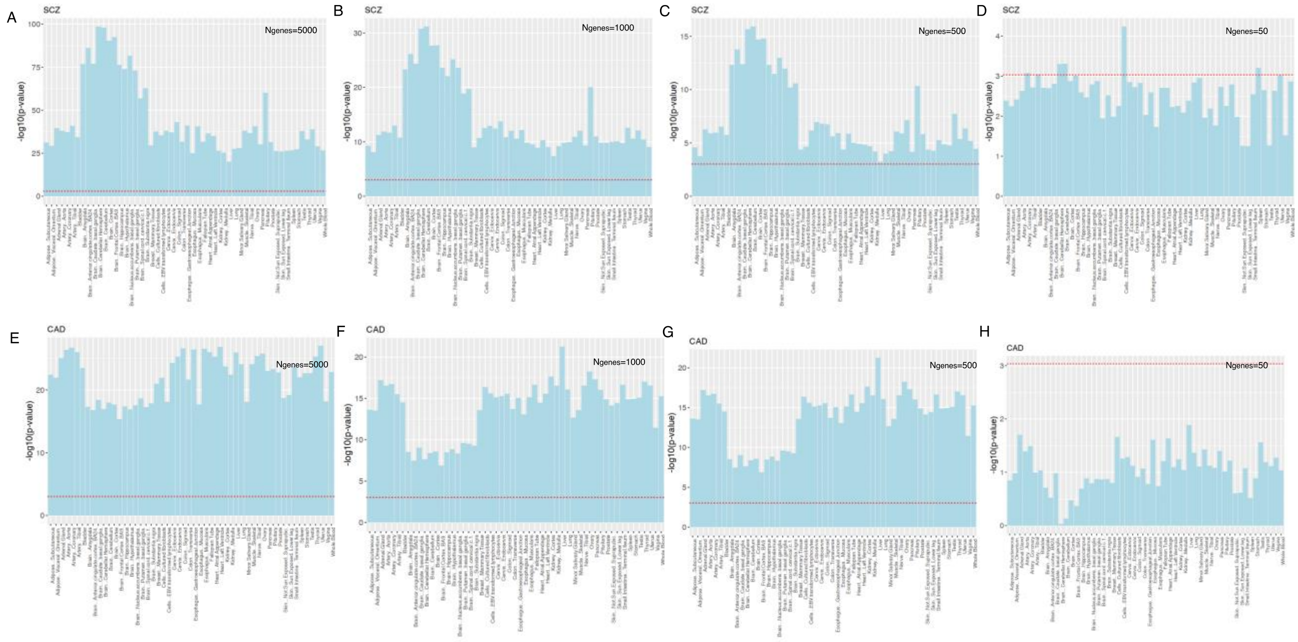

Figure S3. The difference between the expression level of highly and lowly associated genes to schizophrenia (panels A-D), and the coronary artery disease (panels E to H) across 54 tissues using data from the GTEx database. In panels A-D (schizophrenia), and panels E-H (coronary artery disease), we compared 5000, 1000, 500 and 50 highly associated genes with the same number of lowly associated genes. We used MAGMA z-scores to select these genes such that highly and lowly associated genes have the highest and the lowest z-scores. The p-values in all panels were calculated using the Spearman's rank-sum test. The dashed line in all panels correspond to the Bonferroni corrected p-value of 0.05.

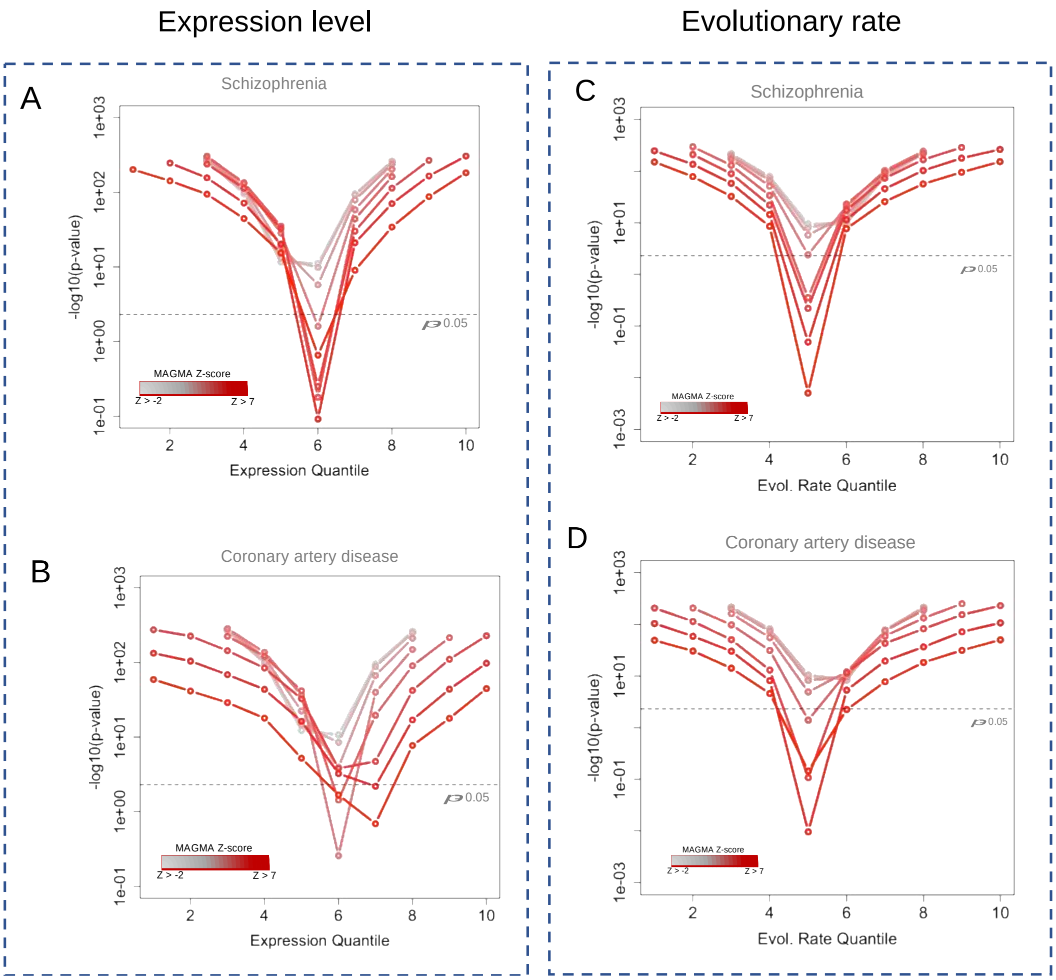

Figure S4. A, B) We calculated the negative base-10 logarithm of p-values to compare the average expression levels of genes that exhibit varying degrees of association with schizophrenia (panel A) and coronary artery disease (panel B) to the average expression levels of human genes in different quantiles of expression. The p values were calculated from Wilcoxon rank sum test. C, D) We calculated the negative logarithm of p-values to compare the evolutionary rate of genes that exhibit varying degrees of association with schizophrenia (panel C) and coronary artery disease (panel D) to the average expression levels of different human genes in different quantiles of evolutionary rate. The p values were calculated from Wilcoxon rank sum test. The color scheme represents genes with MAGMA z-scores greater than a predetermined threshold for their association with each disease. The threshold ranges from -2 (shown in gray) to 7 (shown in red).

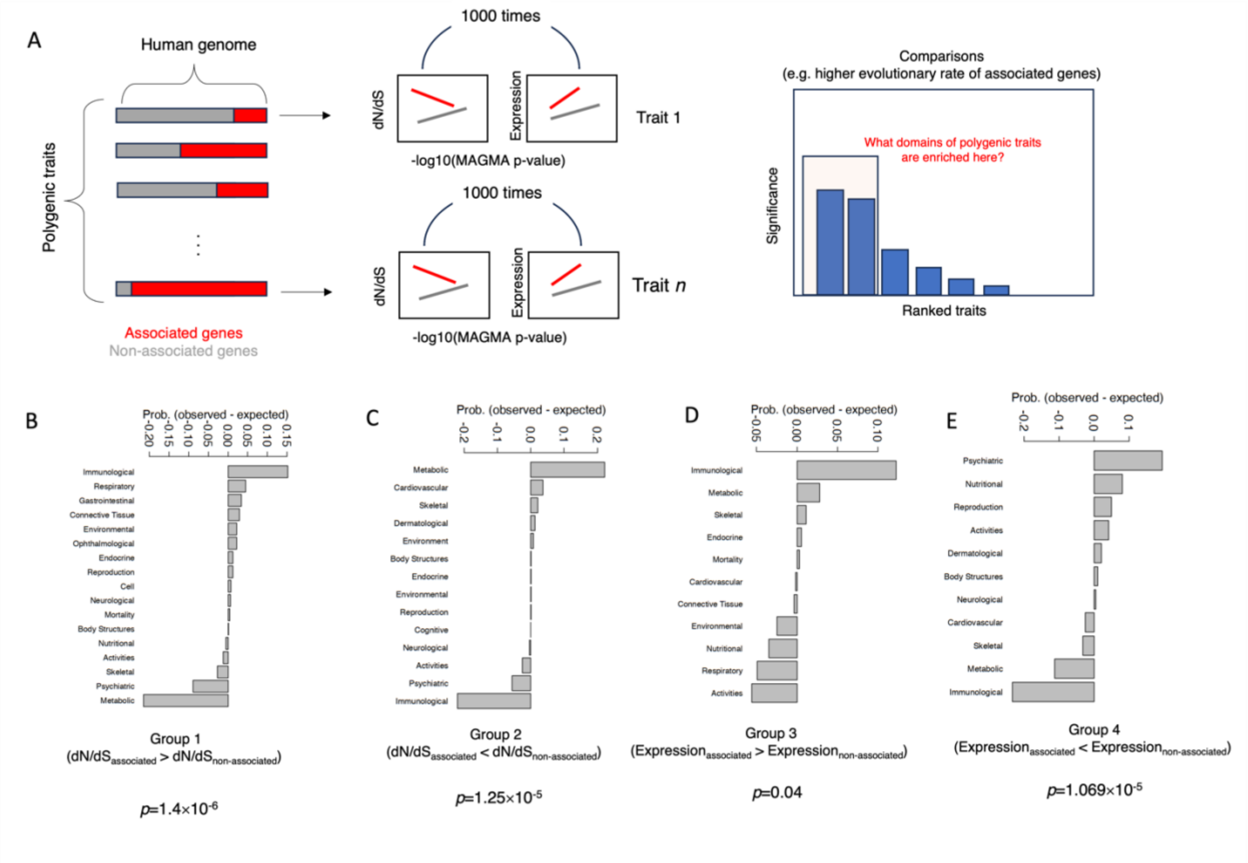

Figure S5. A) The scheme of statistical comparisons of the evolutionary rate and the expression level of associated and non-associated genes with polygenic traits. We selected traits for whom 50 or more associated genes were present (MAGMA p-value < 2.84×10^{-6} as the significance threshold to control for multiple testing). The difference in observed (in foreground sets) and expected proportions (in the background set) for genes whose associated genes have B) a higher rate of evolution, C) a lower rate of evolution, D) a higher expression level, and E) a lower expression level. P-values in panels B to E were calculated using a chi-square test of enrichment.

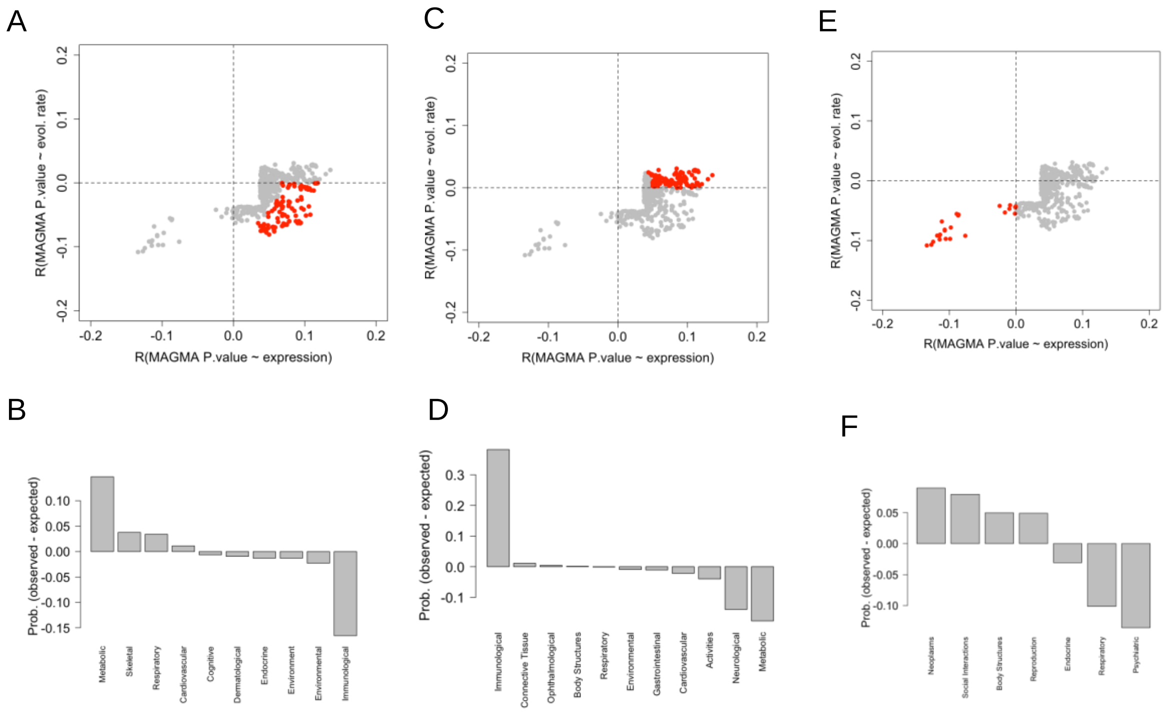

Figure S6. A) Traits for whom the correlation between genetic association and expression level, $R_{exp} > 0$, and the correlation between the genetic association and the evolutionary rate, $R_{rate} < 0$. B) Domains of polygenic traits that are over-represented for the top 100 traits in panel A (shown in red). C) Traits for whom $R_{exp} > 0$ and $R_{rate} > 0$. D) Domains of polygenic traits that are over-represented for the top 100 traits in panel C (shown in red). E) Traits for whom $R_{exp} < 0$ and $R_{rate} < 0$. F) Domains of polygenic traits that are over-represented for the top 100 traits in panel E (shown in red).

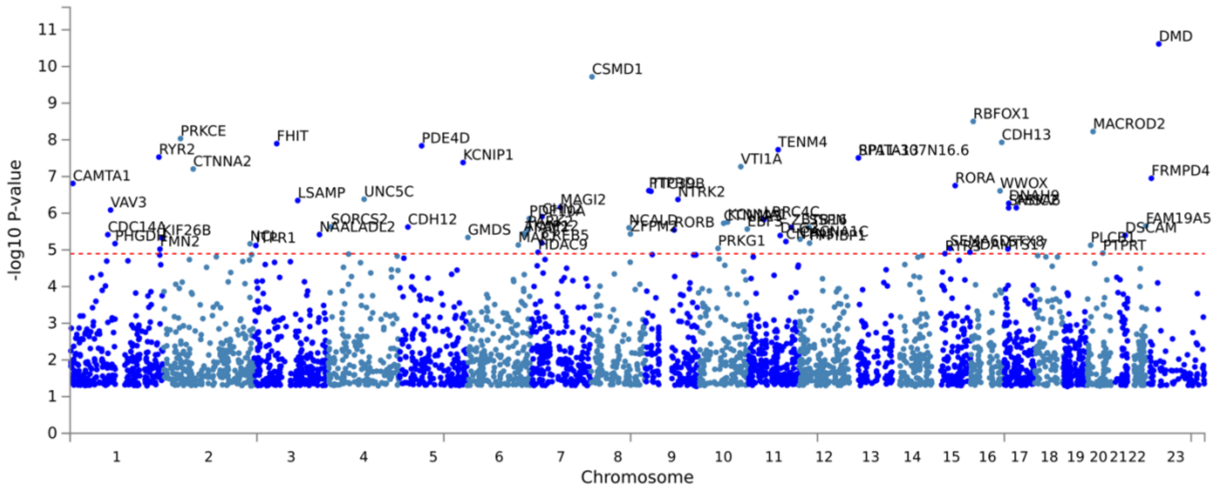

Figure S7. The Manhattan plot showing the gene-based p-value of the association of different genes to squamous carcinoma in lung cancer patients. Input SNPs were mapped to 3917 protein coding genes. Genome wide significance (red dashed line in the plot) was defined at P = 0.05/3917 = 1.276e-5. (Taken from GWAS ATLAS dataset; id=2021).

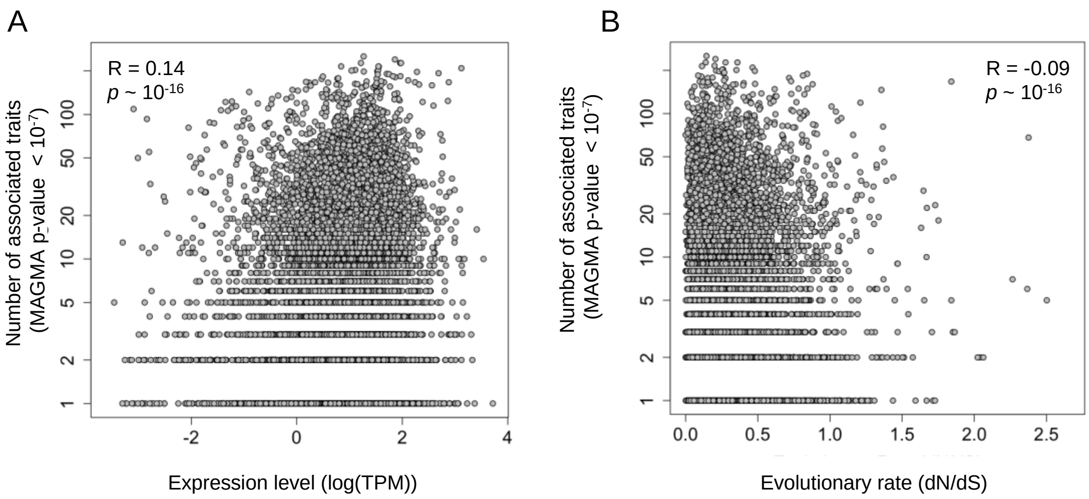

Figure S8. A) The correlation between the number of associated traits per gene (MAGMA p-value > 10^{-7}) and the average expression level. B) The correlation between the number of associated traits per gene (MAGMA p-value > 10^{-7}) and the evolutionary rate.

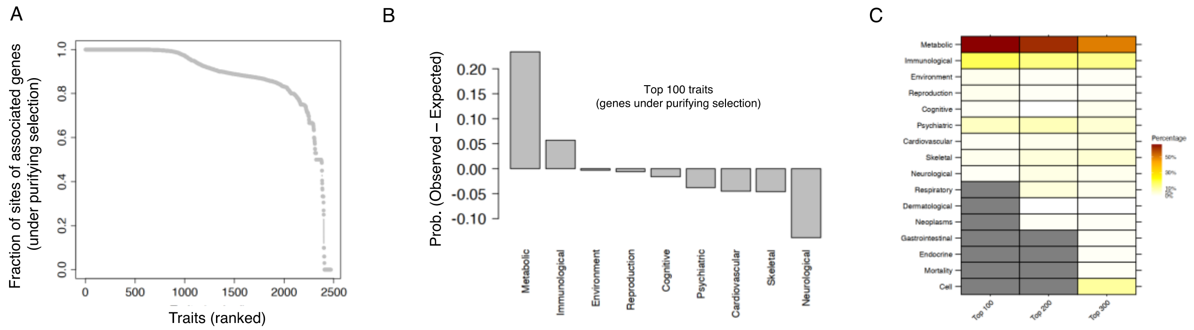

Figure S9. Metabolic traits have likely evolved under purifying selection. A) The fraction of sites in the associated genes to complex traits that evolved under purifying selection. (d*N*/d*S* < 1). B) Observed minus expected probability of different traits in the top 100 traits with the highest fraction of sites under purifying selection. C) Results similar to panel B, extended to the top 200 and 300 traits with the highest fraction of sites under purifying selection.

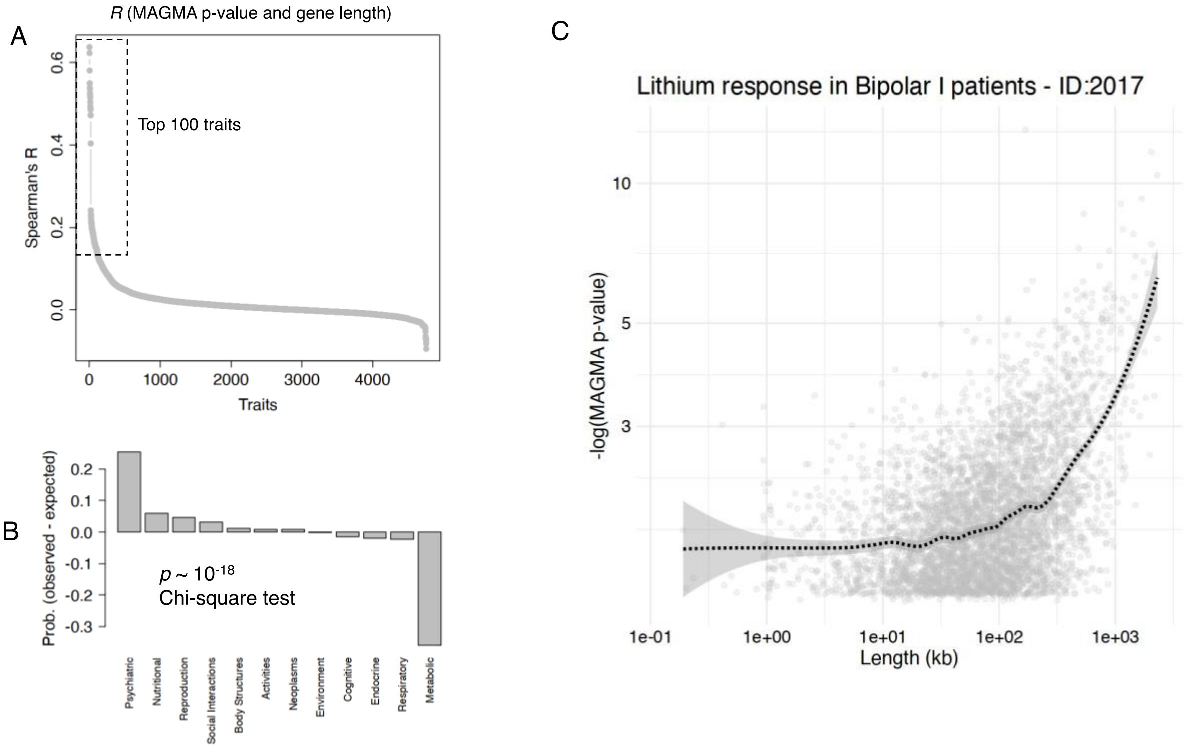

Figure S10. A) The spearman's correlation between gene length and genetic association (-$\log_{10}$(MAGMA p-value)) for 4576 traits within the GWAS ATLAS. B) The enrichment of different domains of polygenic traits within the top 100 traits with the largest gene length bias ($p \sim 10^{-18}$, Chi-square test of enrichment). C) An example of a psychiatric trait, lithium response in bipolar patients (GWAS atlas trait ID: 2017), with a substantial gene length bias (R=0.51, $p<10^{-6}$).

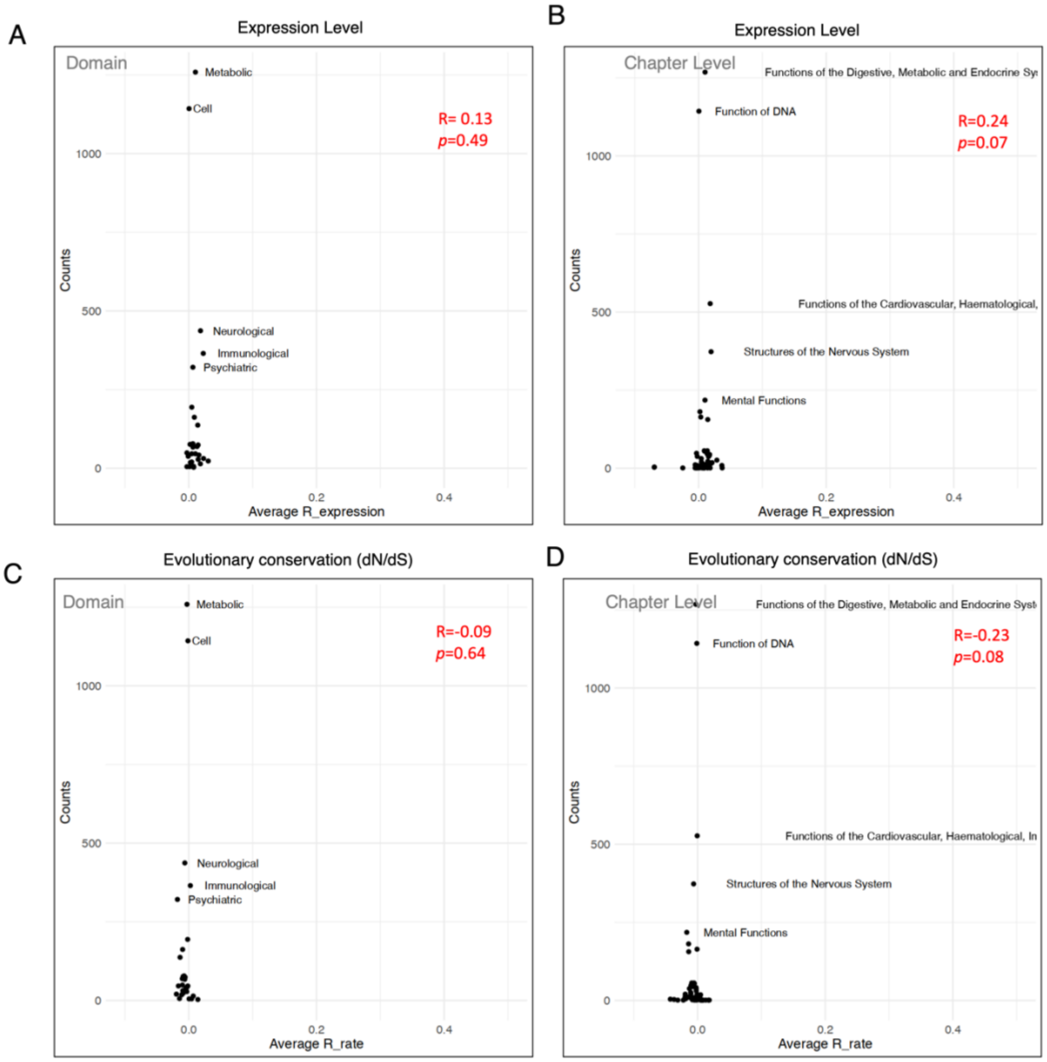

Figure S11. The relationship between the number of GWA studies within domains and subchapters of polygenic traits and the correlation between genetic association and the expression level (panels A and B), and the correlation between genetic association and the evolutionary rate (panels C and D).

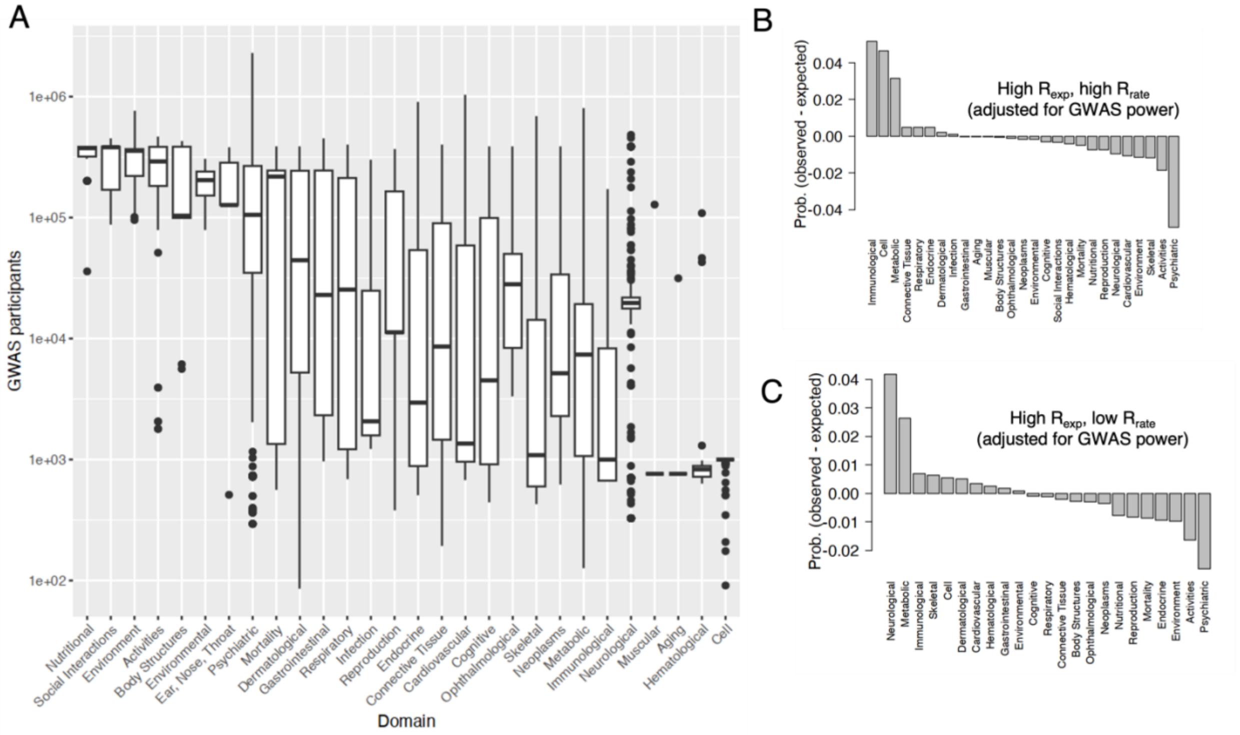

Figure S12. A) The number of GWAS participants for each domain of polygenic traits. The enrichment of different domains of polygenic traits within the residual of a loess regression between R_{exp} and R_{rate} with the GWAS's number of participants for traits with a high R_{exp} and a high R_{rate} (panel B), and a high R_{exp} but a low R_{rate} (panel C).

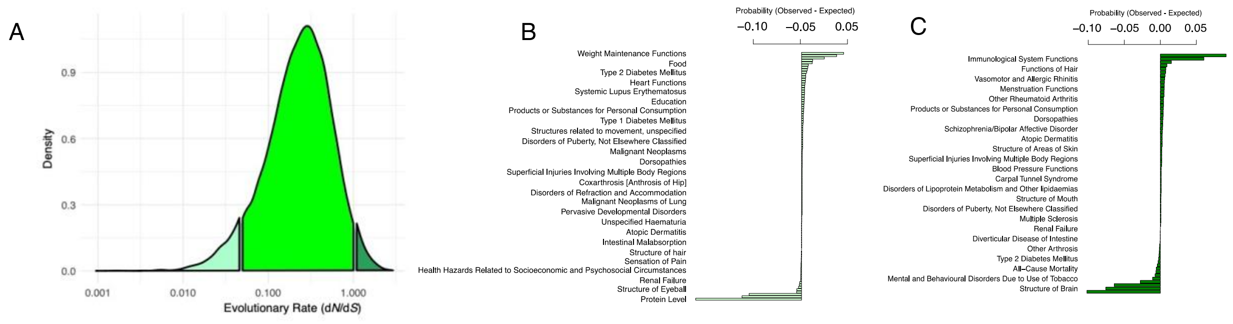

Figure S13. A) The distribution of the evolutionary rate (dN/dS) of human genes The two segments colored in dark and light green correspond to genes evolving with dN/dS>1 and the bottom 10% of genes in evolutionary rates, respectively. B) The overrepresented traits for the bottom 10% of genes in evolutionary rates (light green segment of panel A). C) The overrepresented traits for the genes evolving with dN/dS>1 (dark green segment of panel A). The enrichment was calculated with the chi-square test of enrichment (p-value < 0.05).

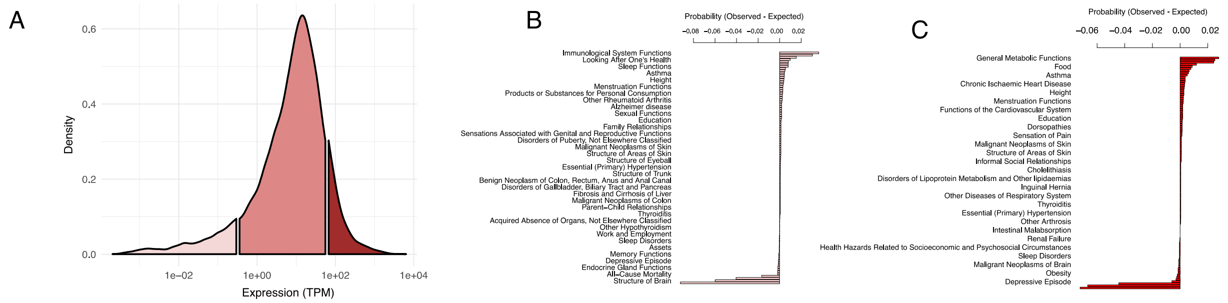

Figure S14. A) The distribution of the average expression level of human genes (in units of transcript per million mapped reads; TPM). The two segments colored in dark and light red correspond to genes at the top and bottom 10% in expression level, respectively. B) The overrepresented traits for the bottom 10% of genes in expression level (light red segment of panel A). C) The overrepresented traits for the bottom 10% of genes in expression level (dark red segment of panel A). The enrichment was calculated with the chi-square test of enrichment (p-value < 0.05).

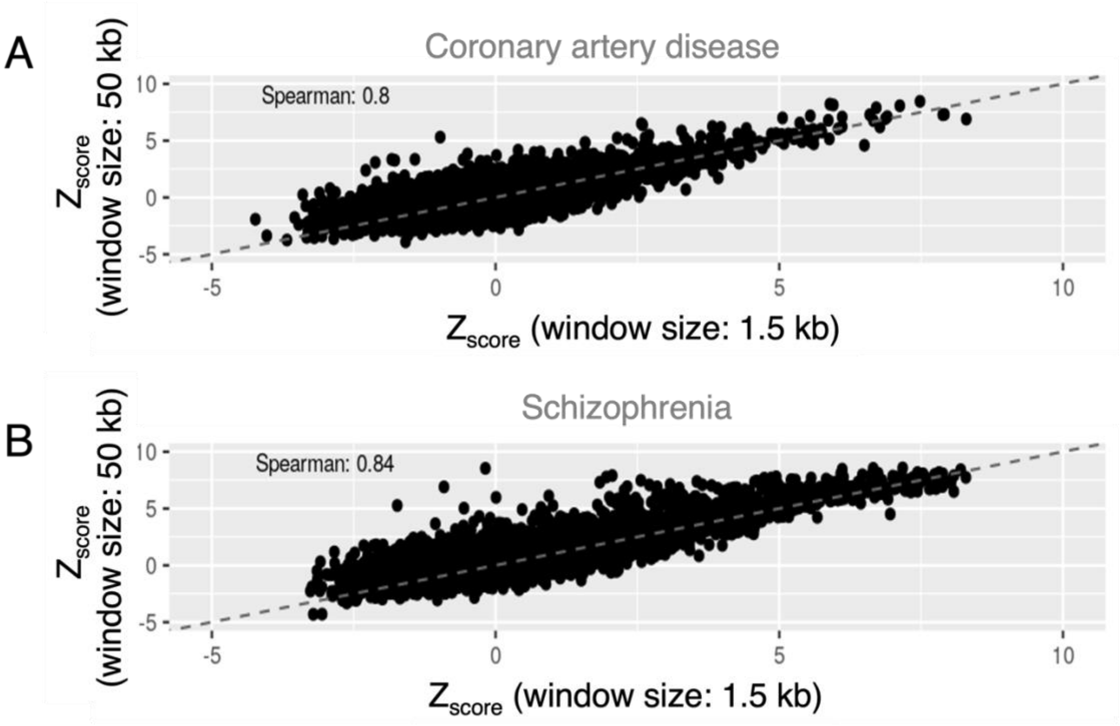

Figure S15. The correlation between calculated MAGMA z-scores with the window size of 50kb (y-axis) versus the MAGMA Z-scores calculated with the window size of 1.5kb for A) the coronary artery disease, and B) Schizophrenia.

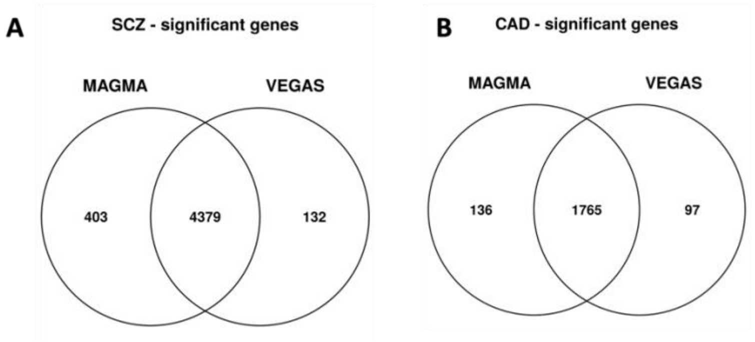

Figure S16. The overlap of MAGMA and VEGAS in genes associated to schizophrenia (panel A, ~89%) and the coronary artery disease (panel B, ~88%).

Supplementary Table 1. The significance ($-\log_{10}$ (p-value)) of the the comparisons of the evolutionary rate and expression level of genes associated with schizophrenia (SCZ), and coronary artery disease (CAD) in different categories of association (MAGMA Z-score bins) with those in different deciles of the corresponding property (gene expression or evolution rate) of 14568 human genes.

	MAGMA Z-score	<Q1	Q1-Q2	Q2-Q3	Q3-Q4	Q4-Q5	Q5-Q6	Q6-Q7	Q7-Q8	Q8-Q9	Q9-Q10
SCZ (Expression level)	Z ≥-2	Inf	Inf	263.70	96.36	11.79	10.98	94.21	261.02	Inf	Inf
	Z ≥ -1	Inf	Inf	273.65	103.98	14.90	8.81	89.56	254.88	Inf	Inf
	Z ≥ 0	Inf	Inf	306.98	127.21	25.06	3.63	70.72	224.79	Inf	Inf
	Z ≥ 1	Inf	Inf	Inf	171.51	48.15	0.34	42.92	173.82	Inf	Inf
	Z ≥ 2	Inf	Inf	Inf	205.16	74.63	7.01	21.74	118.76	Inf	Inf
	Z ≥ 3	Inf	Inf	Inf	176.32	69.35	10.31	10.53	77.22	229.78	Inf
	Z ≥ 4	235.85	292.08	199.09	111.65	37.17	4.00	8.76	50.39	145.44	289.93
	Z ≥ 5	158.50	174.83	117.63	62.02	19.93	1.52	5.07	27.73	83.12	170.83
SCZ (d*N*/d*S*)	Z ≥-2	Inf	Inf	217.59	78.94	9.49	9.69	79.62	218.72	Inf	Inf
	Z ≥ -1	Inf	Inf	212.17	74.86	7.97	11.50	84.26	224.99	Inf	Inf
	Z ≥ 0	Inf	Inf	195.24	65.68	5.76	14.02	90.16	234.28	Inf	Inf
	Z ≥ 1	Inf	Inf	171.78	51.43	2.40	20.78	103.08	243.45	Inf	Inf
	Z ≥ 2	Inf	297.17	129.88	33.84	0.35	23.00	95.45	219.56	Inf	Inf
	Z ≥ 3	Inf	210.43	89.40	22.01	0.22	17.61	73.04	166.96	288.21	Inf
	Z ≥ 4	247.55	135.85	58.19	14.40	0.05	11.43	45.54	102.87	178.58	263.56
	Z ≥ 5	150.69	78.42	32.53	8.55	0.01	7.68	25.71	56.55	95.31	152.62
CAD (Expression level)	Z ≥-2	Inf	Inf	267.09	98.11	12.26	10.74	94.26	261.93	Inf	Inf
	Z ≥ -1	Inf	Inf	271.03	102.88	14.65	8.52	87.89	250.98	Inf	Inf
	Z ≥ 0	Inf	Inf	283.37	116.24	22.44	3.92	66.83	211.28	Inf	Inf
	Z ≥ 1	Inf	Inf	284.86	137.40	36.68	0.26	39.88	149.53	Inf	Inf
	Z ≥ 2	Inf	Inf	224.43	121.98	41.78	1.43	19.58	90.48	214.90	Inf
	Z ≥ 3	275.38	226.13	144.04	84.30	32.64	3.81	4.72	42.14	110.57	228.22
	Z ≥ 4	133.46	104.46	68.76	43.61	16.33	3.24	2.19	16.99	43.90	97.81
	Z ≥ 5	59.19	41.46	29.01	17.95	5.22	1.68	0.69	7.71	17.86	44.69
CAD (d*N*/d*S*)	Z ≥-2	Inf	Inf	217.33	79.18	9.54	9.47	78.56	216.66	Inf	Inf
	Z ≥ -1	Inf	Inf	217.17	81.48	10.45	8.40	74.90	209.83	Inf	Inf
	Z ≥ 0	Inf	Inf	198.74	73.21	8.27	9.71	75.61	204.93	Inf	Inf
	Z ≥ 1	Inf	Inf	161.48	55.38	4.92	12.06	75.67	186.66	Inf	Inf
	Z ≥ 2	Inf	207.86	98.20	31.37	1.40	11.57	59.58	132.54	251.57	Inf
	Z ≥ 3	207.43	114.12	50.56	13.00	0.11	11.51	42.98	82.13	153.87	230.51
	Z ≥ 4	104.13	58.91	30.25	8.13	0.01	5.33	19.54	36.64	71.77	107.16
	Z ≥ 5	49.10	30.53	14.17	4.58	0.14	2.23	7.75	18.36	31.47	50.19

Supplementary Table 2. The bimodality coefficient of d*N*/d*S* distribution for genes linked to monogenic diseases and the highly associated genes with schizophrenia and the coronary artery disease.

	**Bimodality Coefficient of dN/dS**		
**Diseases**	**MAGMA_Z > 4**	**MAGMA_Z > 5**	**MAGMA_Z > 6**
SCZ	0.56	0.63	0.65
CAD	0.55	0.54	0.58
Monogenic	0.44		

Supplementary Note 1. Calculation of bimodality coefficient

To assess whether the evolutionary rate distributions of genes linked to polygenic disorders like schizophrenia and coronary artery disease exhibit bimodality, we computed the bimodality coefficient (BC)[1-3]. This coefficient, denoted as BC, incorporates factors such as sample size (n), skewness (m3), and excess kurtosis (m4) of the distribution under examination:

$$BC = \frac{m_2^2 + 1}{m_4 + 3\frac{(n-1)^2}{(n-2)(n-3)}}$$

Here, m3 represents skewness and m4 denotes excess kurtosis. The BC of a specific empirical distribution is then contrasted with a reference value, $BC_{crit} = 5/9 \approx 0.555$, indicative of a uniform distribution[4]. Higher BC values suggest bimodality, while lower values indicate unimodality.

References

1 DeCarlo, L. T. On the meaning and use of kurtosis. *Psychological methods* **2**, 292 (1997).
2 Joanes, D. N. & Gill, C. A. Comparing measures of sample skewness and kurtosis. *Journal of the Royal Statistical Society: Series D (The Statistician)* **47**, 183-189 (1998).
3 Pfister, R., Schwarz, K. A., Janczyk, M., Dale, R. & Freeman, J. Good things peak in pairs: a note on the bimodality coefficient. *Frontiers in psychology* **4**, 67498 (2013).
4 Freeman, J. B. & Dale, R. Assessing bimodality to detect the presence of a dual cognitive process. *Behavior research methods* **45**, 83-97 (2013).
